# Modulatory actions of *Echinococcus granulosus* antigen B on macrophage inflammatory activation

**DOI:** 10.3389/fcimb.2024.1362765

**Published:** 2024-03-18

**Authors:** Ana Maite Folle, Sofía Lagos Magallanes, Martín Fló, Romina Alvez-Rosado, Federico Carrión, Cecilia Vallejo, David Watson, Josep Julve, Gualberto González-Sapienza, Otto Pristch, Andrés González-Techera, Ana María Ferreira

**Affiliations:** ^1^ Unidad de Inmunología, Instituto de Química Biológica, Facultad de Ciencias, Universidad de la República, Montevideo, Uruguay; ^2^ Área Inmunología, Departamento de Biociencias, Facultad de Química, Universidad de la República, Montevideo, Montevideo, Uruguay; ^3^ Unidad de Biofísica de Proteínas, Institut Pasteur, Montevideo, Uruguay; ^4^ Institute of Pharmacy and Biomedical Sciences, University of Strathclyde, Glasgow, United Kingdom; ^5^ Research group of Endocrinology, Diabetes and Nutrition, Institut de Recerca SANT PAU, Barcelona, Spain; ^6^ Centro de Investigación Biomédica en red de Diabetes y Enfermedades Metabólicas asociadas, Instituto de Salud Carlos III, Madrid, Spain

**Keywords:** cestodes, Echinococcus granulosus, HLBP family, lipoprotein, antigen B, immunomodulation, macrophage

## Abstract

Cestodes use own lipid-binding proteins to capture and transport hydrophobic ligands, including lipids that they cannot synthesise as fatty acids and cholesterol. In *E. granulosus* s.l., one of these lipoproteins is antigen B (EgAgB), codified by a multigenic and polymorphic family that gives rise to five gene products (EgAgB8/1-5 subunits) assembled as a 230 kDa macromolecule. EgAgB has a diagnostic value for cystic echinococcosis, but its putative role in the immunobiology of this infection is still poorly understood. Accumulating research suggests that EgAgB has immunomodulatory properties, but previous studies employed denatured antigen preparations that might exert different effects than the native form, thereby limiting data interpretation. This work analysed the modulatory actions on macrophages of native EgAgB (nEgAgB) and the recombinant form of EgAg8/1, which is the most abundant subunit in the larva and was expressed in insect S2 cells (rEgAgB8/1). Both EgAgB preparations were purified to homogeneity by immunoaffinity chromatography using a novel nanobody anti-EgAgB8/1. nEgAgB and rEgAgB8/1 exhibited differences in size and lipid composition. The rEgAgB8/1 generates mildly larger lipoproteins with a less diverse lipid composition than nEgAgB. Assays using human and murine macrophages showed that both nEgAgB and rEgAgB8/1 interfered with *in vitro* LPS-driven macrophage activation, decreasing cytokine (IL-1β, IL-6, IL-12p40, IFN-β) secretion and ·NO generation. Furthermore, nEgAgB and rEgAgB8/1 modulated *in vivo* LPS-induced cytokine production (IL-6, IL-10) and activation of large (measured as MHC-II level) and small (measured as CD86 and CD40 levels) macrophages in the peritoneum, although rEgAgB8/1 effects were less robust. Overall, this work reinforced the notion that EgAgB is an immunomodulatory component of *E. granulosus* s.l. Although nEgAgB lipid’s effects cannot be ruled out, our data suggest that the EgAgB8/1 subunit contributes to EgAgB´s ability to regulate the inflammatory activation of macrophages.

## 1 Introduction

Cystic echinococcosis (CE) is a cosmopolitan zoonosis caused by the larval stage of cestodes belonging to the species complex *Echinococcus granulosus* sensu lato (s. l.). One of the best-characterised antigens of this organism is the so-called antigen B (EgAgB), a highly antigenic lipoprotein that was previously identified as a member of the cestode-specific family of hydrophobic ligand binding proteins (HLBPs, reviewed by Silva-Álvarez et al., 2015a). Complex gene subfamilies that have undergone independent expansion events in distinct cestode lineages encode the members of the HLBP family giving rise to species and gene-specific monophyletic clades (Saghir et al., 2000; Kim et al., 2011). The EgAgB subfamily of *E. granulosus* s.l. comprises five clades called *EgAgB1*–*EgAgB5* (reviewed by Olson et al., 2012). These clade´s expression patterns differ in parasite life cycle stages and distinct target tissues at a given developmental stage. The mature protein products of the EgAgB genes were initially characterised as small (8 kDa) α-helix-rich polypeptides, referred to as the EgAgB8/1 to EgAgB8/5 subunits. The subunits could oligomerize and bind miscellaneous fatty acids (FAs) in their holo (Chemale et al., 2005; Monteiro et al., 2012) and apo (Silva-Álvarez et al., 2015b) conformations. In the larval stage, the most expressed gene is *EgAgB1* followed by *EgAgB3*, which is seemingly over-represented in the protoscolex (Zhang et al., 2010). Concomitantly, EgAgB8/1 is the most abundant subunit in EgAgB purified from hydatid fluid (HF) collected from either fertile or infertile hydatids (Silva-Álvarez et al., 2016; Folle et al., 2017). Our group made substantial progress in biochemically characterising the native form of EgAgB purified from HF, revealing that it is a large 230 kDa lipoprotein, of which approximately one-half of its total mass corresponds to lipids, both neutral and polar classes (Obal et al., 2012). Notably, the EgAgB subunit´s lipid-binding abilities may enhance their oligomerization, contributing to the formation of large lipoprotein complexes (Silva-Álvarez et al., 2015b). Based on these observations, we propose that EgAgB may adopt a structural organisation similar to the circulating high-density lipoproteins (HDL) in vertebrates, with approximately one dozen EgAgB8 subunits embedded in an outer hydrophilic phospholipid layer surrounding the lipoprotein´s hydrophobic lipid core (Obal et al., 2012).

The role EgAgB plays in host-parasite immunobiology has not been completely elucidated. Similarly to other *Taenia* antigens belonging to the HLBP family, EgAgB is an immunodominant antigen (Saghir et al., 2000). It is the most genus-specific *E. granulosus* antigen for serodiagnostic tests (reviewed by Mamuti et al., 2006; Siracusano et al., 2008). Most CE patients bearing active/transitional hydatids (CE1 to CE3) develop strong anti-EgAgB IgG responses, with a predominance of the IgG4 subclass, indicating that native EgAgB reaches host tissues to be processed by innate antigen-presenting cells. How and when EgAgB crosses the hydatid wall is uncertain. It might occur before and/or after the generation of miniature hydatids, and be limited by the thickening of the laminar layer and favoured by the protoscolex development. On the other hand, several studies suggest that EgAgB might be involved in immunoregulation. EgAgB binding and internalisation by macrophages via caveolae/raft-mediated endocytosis have been demonstrated *in vitro* (Silva-Álvarez et al., 2016; Da Silva et al., 2018). *In vitro* EgAgB interactions with myeloid cells inhibited the ROS generation and the chemotactic responses of activated neutrophils by pathogen-associated molecular patterns (PAMPs) (Shepherd et al., 1991; Riganò et al., 2001), in addition to interfering with the differentiation, maturation and activation of dendritic cells by lipopolysaccharide (LPS) (Riganò et al., 2007). Interestingly, a recent study showed that EgAgB controlled intestinal inflammation development in a murine bowel disease model by favouring the differentiation of M2-like peritoneal macrophages (Bao et al., 2022). However, most studies have used EgAgB preparations obtained by electroelution from SDS-PAGE gels (Shepherd et al., 1991; Riganò et al., 2001) or differential precipitation methods, including heating to 100°C (Riganò et al., 2007; Bao et al., 2022). Although these preparations allowed researchers to describe some putative EgAgB immunomodulatory properties, denaturing methods for EgAgB purification likely altered the lipoprotein composition and structure, making them sub-optimal for studying how EgAgB interacts with innate immune cells in the physiological conditions during hydatid infection.

Our research group has previous experience in purifying native EgAgB (nEgAgB) by using chromatography, based on the EB7 murine monoclonal antibody (mAb) affinity for EgAgB8/1 (González et al., 1996). Moreover, we proved that nEgAgB interferes with the THP-1 macrophage cytokine response to pro-inflammatory stimuli *in vitro* (Silva-Álvarez et al., 2016). Unfortunately, long-term EB7-secreting hybridoma storage caused an irreversible lack of mAb specificity, leading to the seeking of alternatives for native EgAgB purification. Using a conventional ultracentrifugation method for purifying plasma lipoproteins while preserving the lipoprotein structure (Chapman et al., 1981), we obtained a high-purity native EgAgB preparation (referred to as low-density fraction, Ld_f_) (Folle et al., 2017). However, applying this methodology to several HF samples yielded variable Ld_f_ endotoxin levels, masking *in vitro* EgAgB immunomodulatory activity (**Supplementary Figure S1**). Endotoxin depletion by polymixin B-agarose failed because EgAgB bound to this matrix with a high affinity. Immunoaffinity chromatography seems to be an essential step in purifying nEgAgB, in amounts and purities appropriate for immunological studies.

In this paper, we analysed the potential of both native EgAgB (nEgAgB) and the recombinant form of EgAg8/1 (rEgAgB8/1) to modulate macrophage inflammatory activation. Studies on rEgAgB8/1, the most abundant subunit present in *E. granulosus* s.l. HF (Monteiro et al., 2012; Silva-Álvarez et al., 2016; Folle et al., 2017), allows us to evaluate its involvement in EgAgB effects and potential as a model for investigating EgAgB immunomodulatory properties; this is relevant as the availability of fertile parasite material might limit nEgAgB studies. rEgAgB8/1 was expressed in *Drosophila melanogaster* S2 cells, avoiding eventual contamination associated with bacterial and yeast expression systems that might activate innate immune cells. For purifying nEgAgB and rEgAgB8/1 by immunoaffinity chromatography, we developed an anti-EgAgB8/1 nanobody (the recombinant fragment derived from the variable domain of camelid heavy-chain-only antibodies, referred to as VHH (Vincke and Muyldermans, 2012). Overall, this work provided new methodological tools for EgAgB biology research and generated novel data supporting the EgAgB´s immunomodulatory potential as revealed from *in vitro* and *in vivo* functional assays.

## 2 Materials and methods

### 2.1 Parasite material

Fertile hydatids were collected from the livers and lungs of naturally infected cows during the routine work of local abattoirs in Montevideo, Uruguay. The hydatid content was aspirated under aseptic conditions to separate the fluid and protoscoleces. HF was preserved by the addition of 5 mM EDTA and 20 µM 3,5-di-tert-butyl-4-hydroxytoluene (BHT) at -20°C until use. Protoscoleces were used for hydatid genotyping, performed by amplification and sequencing of a fragment of the mitochondrial cytochrome c oxidase subunit 1 (COX1) (Cucher et al., 2011). The sequencing reactions were carried out at Macrogen (Korea), finding that parasite material belonged to *E. granulosus s.s.* (G1 genotype) and *E. ortleppi* (G5 genotype). Because of limitations in the availability of fertile hydatids and the minimal HF volume needed for obtaining enough amounts for studies, HF samples were pooled, obtaining pools with a G1/G5 ratio between 100/0 and 78/22 (expressed in volume).

### 2.2 VHH/VHs library construction

An adult (4 years-old) female llama (*Lama glama*) from the Montevideo municipal Zoo (Parque Lecocq, Montevideo, Uruguay) was immunised by subcutaneous administration (priming and booster) of 400 µg (protein) of EgAgB emulsified in incomplete Freund´s adjuvant (Sigma-Aldrich, USA). For this purpose, the EgAgB-enriched fraction called Ld_f_, with a purity around 98%, was prepared from fertile HF using the methodology based on Q-Sepharose (GE Healthcare, Life Sciences, Sweden) ion exchange chromatography followed by ultracentrifugation on a KBr gradient (Folle et al., 2017). Twenty-one days after the last booster 150 mL of blood was collected in double blood collection bags containing sodium citrate as anticoagulant. All animal manipulation was performed by veterinarians of the Zoo under strict animal welfare guidelines of the Ethical Committee of the zoo.

A phage display library of VHHs/VHs (the heavy chain variable domain of conventional antibodies) was constructed as previously described (Rossotti et al., 2015). Briefly, peripheral blood mononuclear cells were obtained from collected blood (150 mL) by centrifugation on Histopaque-1077 (Sigma-Aldrich) gradients according to the manufacturer’s recommendations. Total RNA was extracted using TRIZOL reagent (Invitrogen, Carlsbad, CA, USA) from about 10^7^ cells, and quantified by measuring the absorbance at 260 nm in a Nano Drop (Thermo Fisher Scientific, Fremont, CA) and reverse transcribed using superscript III first-strand synthesis system for RT-PCR (Invitrogen) with random hexamers. The genes encoding both VHH and VH were then PCR-amplified using the obtained cDNA as template and the previously described set of forward and reverse primers (Rossotti et al., 2015). The amplified DNA was digested taking advantage of the SfiI sites introduced with the primers and cloned in the phagemid pComb3X vector (generously donated by Professor Barbas, The Scripps Research Institute, La Jolla, USA). This vector encodes an in-frame 6xHis tag downstream of the cloning site, followed by the encoding sequence of a hemagglutinin epitope (HA), a stop amber codon, and a truncated version of the pIII coat protein of M13. The expression of the inserted gene is under the control of the lacZ promoter, and after induction with 1mM isopropyl-β-d-thiogalactopyranoside (IPTG, Sigma-Aldrich) an important proportion of the recombinant protein is secreted to the media as VHH-6xHis-HA recombinant protein. The ligated vector was electroporated into highly competent ER2738 *Escherichia coli* cells (Lucigen Corporation, WI). The cells were cultured and the phagemid-borne phage library displaying the VHH/VH repertoire was rescued by superinfection with the M13KO7 helper phage (Pharmacia Biotech, Uppsala, Sweden) and cultured overnight (ON) in the presence of 40 μg/mL of kanamycin. The phages were precipitated with 0.2 volumes of 20% polyethylene glycol 8000, 2.5 M NaCl, on ice during 1 hour, centrifuged and afterwards the collected phages were resuspended in phosphate-buffered saline (PBS) to a final titer of 10^12^ cfu/mL.

### 2.3 Panning of the VHH/VHs library and screening of anti-EgAgB clones

Microtiter high binding ELISA plates (Maxisorp, Nunc, USA) were coated ON at 4°C with 100 μL/well of 10 μg/mL EgAgB (Ld_f_) in PBS. After coating, wells were blocked with 300 μL/well of 3% bovine serum albumin (BSA, Sigma-Aldrich) in PBS. The antibody library (10^12^ transducing units) was then mixed with PBS containing 0.05% Tween 20 and 3% BSA, and dispensed into the plates. After incubating for 1 hour at 4°C, plates were washed extensively (8 times) with PBS containing 0.05% Tween 20 (PBS-T), after which the bound phages were eluted by incubation at 37°C with 100 μL/well of 10 mg/ml trypsin (Sigma-Aldrich). The eluted phages (output) were immediately neutralised by adding 100 μL/well of 100 mg/ml trypsin inhibitor (Sigma-Aldrich). The obtained phages were amplified and used for one additional round of panning, but employing ELISA plates coated with 100 μL/well of 0.2 μg/mL EgAgB. After the second round, 10-fold serial dilutions of the phage output (100 μL) were used to infect a fixed amount (100 μL) of ER2738 *E. coli* cells (0.5 x 10^8^ cells/mL). After infection, cells were plated on ampicillin plates and colony counting allowed assessing the number of phage particles obtained.

### 2.4 Cloning of VHH/VHs DNAs coding sequences in a robust expression system

For VHHs/VHs expression, the coding sequences from the second output present in pCOM3X vector were cloned *en masse* into the pINQ-H6HA vector, a modified version of pET28a+ (Novagen) which includes an N-terminal OmpA leader peptide sequence for directing the VHHs/VHs to the periplasmic space and C-terminal HA epitope coding sequence. This vector allows the highly efficient expression of genes under a T7 RNA polymerase promoter when incorporated into inducible T7 RNA polymerase expressing cells, such as *E. coli* BL21 strain. To that end, plasmid DNA from cells infected with phages from the second output was prepared by performing a miniprep (QIAGEN). The plasmids containing the pool of VHHs/VHs genes were PCR amplified with primers Fw: 5´-GTTACTCGCGGCCCAGGCGGCCATG-3´and Rv: 5´-CCACGATTCTGGCCGGCC TGGCCTGAG-3´, annealing outside SfiI restriction sites. The PCR product was electrophoresed in an agarose gel, gel extracted (QIAGEN), and digested with SfiI restriction enzyme. The digested and purified PCR product was ligated into pINQ-H6HA vector (previously digested with SfiI and purified) with T4 DNA Ligase (Thermo Scientific), and the ligation mix was electroporated into BL21 *E. coli* electrocompetent cells. The transformants were plated on LB agar plates containing 40 µg/mL kanamycin and 4% glucose and incubated ON at 37°C. The next day, 10 individual clones were tested to check that anti-EgAgB VHHs/VHs were successfully expressed from the pINQ-H6HA vector. For this, the same methodology described above for the screening of positive clones was used except that induction of VHHs/VHs expression was performed at an OD_600_ equals to 0.5, with 3 μM IPTG (final concentration). After induction, cultures were incubated for 4 hours at 37°C, with shaking (250 rpm) to allow VHHs expression. One mL of culture was centrifuged, the supernatant was discarded, and the cells in the pellet were resuspended in PBS (500 μL), followed by lysis by three cycles of freeze/thaw with sonication. After centrifuging at 17,000 x *g*, 100 µL of the supernatant were transferred to wells of an ELISA plate coated with the EgAgB fraction, Ld_f_, (0.2 μg/well) and blocked with BSA (1%). As a control, samples were also transferred to BSA-coated wells. VHH binding was detected by incubation for 1 hour at room temperature (RT) with anti-HA-peroxidase (Roche, 1/3,000 dilution) and the peroxidase activity developed using TMB/H_2_O_2_ substrate and analysed spectrophotometrically at 450 nm.

### 2.5 Sequencing of DNAs coding anti-EgAgB VHHs/VHs clones

Plasmids from EgAgB positive clones expressed from pINQ-HAH6 were isolated by performing minipreps (QIAGEN). Their sequences were obtained by Sanger sequencing by Macrogen (Korea) and manually aligned.

### 2.6 EgAgB subunit recognition of anti-EgAgB VHHs/VHs clones

EgAgB positive VHHs/VHs clones (n=9) were evaluated by ELISA for their binding to purified delipidated recombinant EgAgB subunits, named drEgAgB8/1, drEgAgB8/2 and drEgAgB8/3 (*E. coli*-expressed subunits (Silva-Álvarez et al., 2015b), generously donated by Dr Valeria Silva-Álvarez, Área Inmunología, Facultad de Química, UdelaR). For this purpose, ELISA plates were coated with each of the purified drEgAgB8 subunits at 0.5 μg/well and blocked as mentioned above. Then, the supernatant of cultures expressing anti-EgAgB VHHs/VHs was incubated at 1:5 dilution (1 hour, RT with agitation) and VHH binding was developed as previously described.

### 2.7 Expression of anti-EgAgB VHHs/VHs clones in BL21 *E. coli* cells

The expression of anti-EgAgB VHHs/VHs clones showing the highest binding ability to rEgAgB8/1 (named clones 1, 3 and 7) was analysed in 10 mL LB cultures as described above. After allowing expression, 1 mL of the respective cultures was centrifuged at 5,000 x *g* for 10 min. The supernatant was discarded and the pellet was resuspended in 100 μL of B-PER reagent (Thermo Fisher Scientific), vortexed, and incubated at RT for 15 min to allow *E. coli* membrane disruption. After adding PBS (200 μL), the soluble protein fraction was separated by centrifugation for 10 min at 5,000 x *g*. The remaining pellet was extracted with 8 M urea (30 μL) during 15 min at RT. After PBS addition (270 μL), the supernatant corresponding to the insoluble fraction was separated by centrifugation for 5 min at 17,000 x *g*. Twenty μL of both soluble and insoluble fractions were loaded on 15% SDS-PAGE gels, run, and stained with Coomassie Blue as detailed below.

### 2.8 Preparation of anti-EgAgB immunoaffinity column

The anti-EgAgB VHH clone 1 (for simplicity anti-EgAgB clone 1) was expressed in 500 mL of liquid LB medium by IPTG induction as described above. After 4 hours induction, the culture was centrifuged at 6,000 x *g* at 4°C for 15 min. The supernatant was discarded and the cellular pellet was washed using LB medium (25 mL), centrifuged as mentioned above and suspended in PBS (10 mL). The cells were then lysed with 0.2 M Tris-HCl pH 8.0 containing 0.5 mM EDTA and 0.5 M sucrose. The lysed cells were then centrifuged at 17,000 x *g* at 4°C for 20 min and the supernatant was filtered through 0.45 μM pore size filters. VHH purification was performed on Ni-NTA columns in the ÄKTA purification system (General Electric Healthcare, Uppsala, Sweden) according to the manufacturer’s instructions. The purified anti-EgAgB clone 1 was covalently bound to CNBr-Sepharose (GE Healthcare) using a ratio of 10 mg protein/mL matrix and following the protocol recommended by the manufacturer. The efficiency of coupling was around 99% and was estimated by measuring the absorbance at 280 nm of the liquid phase after conjugation. To avoid any possible contamination between native and recombinant EgAgB samples, the obtained anti-EgAgB clone 1-Sepharose matrix was divided into two immunoaffinity columns (1.5 mL each).

### 2.9 Purification of EgAgB from HF

EgAgB was prepared from pooled HF taking advantage of the previously described methodology based on anion exchange chromatography followed by ultracentrifugation in a KBr density gradient (Folle et al., 2017), and adding a final step of affinity chromatography on anti-EgAgB clone 1-Sepharose. All buffers were prepared using pyrogen-free water (ICU-VITA, Uruguay). Briefly, HF from hydatids was firstly clarified by centrifugation (10,000 x g for 20 min, 4°C) and filtration through 0.45 µm filter membranes (Millipore). For the whole work, we prepared EgAgB batches (biological replicates, n=5) using between 7 and 20 hydatids each. The clarified HF was then fractionated by anion exchange chromatography on Q-Sepharose equilibrated in 20 mM phosphate buffer, pH 7.4 containing 200 mM NaCl, 5 mM EDTA and 20 µM BHT. The retained fraction containing EgAgB (QS_f_), was eluted by changing ionic strength to 500 mM NaCl in a single step. After concentration (~10-fold) and equilibration in PBS containing 5 mM EDTA and 20 µM BHT (PBS_EB_) for preventing lipid oxidation, QS_f_ was ultracentrifuged in a KBr gradient (4 hours at 332,000 x g), obtaining Ld_f_, a yellowish-brown band of lower density enriched in EgAgB. Ld_f_ was equilibrated in PBS_EB_ and applied on the anti-EgAgB clone 1-Sepharose, previously equilibrated in PBS_EB_. After washing with PBS_EB_ and 0,1M glycine buffers at pH 5 and 4 (2 column volumes each), EgAgB was eluted using 0.1 M glycine pH 2.5. Using a PD-10 desalting column (GE Healthcare, USA) the eluted fraction was equilibrated in PBS_EB_ plus antibiotic/antimycotic (100 U/mL penicillin, 0.1 mg/m streptomycin and 250 ng/mL amphotericin B, PBS_EBAb_) to prevent further contamination. Immunopurified nEgAgB was maintained (for a maximum of 2 months) at 4°C under a N_2_ atmosphere. Analysis of the endotoxin level was carried out using a chromogenic *Limulus amebocyte* lysate assay (LAL assay with a cut-off of 0.03 UE/mL, Beltran Zunino, Montevideo).

### 2.10 Obtaining rEgAgB8/1 expressed in eukaryotic insect cells

The EGR_06805 sequence corresponding to EgAgB8/1 subunit (https://parasite.wormbase.org) was optimised for expression in *D*. *melanogaster* using GenScript software. Afterwards, we designed and acquired from GenScript a construct (EgAgB8/1 construct, **Supplementary Figure S2**), containing the optimised EGR_06805 sequence flanked by the sequences to be used as forward and reverse primers for hybridization with the expression vector. Then, we cloned the optimised EGR_06805 sequence, by restriction free (RF)-cloning, in a modified version of the pMT/BiP/V5-His vector, called pDroEx (Ortega et al., 2018). This vector included two tandem sequences for Streptavidin binding (Strep-Tag) and two DNA fragments, flanking the insert gene, to be used for hybridization with generic forward and reverse primers during RF-cloning (Van Den Ent and Löwe, 2006). For Rf-cloning, we used DNA polymerase Phusion, 0.5 µM of generic primers (first PCR) or 100 ng of the EgAgB8/1 construction (second PCR), 20 ng vector and the following conditions: a denaturing step at 98°C for 30 s, 30 amplification cycles (98°C for 10 s, 65°C for 30 s and 72°C for 1 min), and a final extension step at 72°C for 5 min. PCR products were then treated with 20 U of DpnI (Thermo) for 90 min at 37°C followed by 20 min at 80°C to selectively degrade the methylated parental vector. The digestion products were then transformed in competent *E. coli* XL1 and EgAgB8/1 presence was confirmed by colony-PCR. Positive clones were expanded in LB cultures supplemented with ampicillin (100 µg/ml) and incubated ON at 37°C. Plasmids (pDroEx-EgAgB8/1) were purified using the QIAfilter Plasmid Midi Kit (Qiagen, Germany) and sequenced (Macrogen, Korea) for verification (**Supplementary Figure S2**).

A stably transformed *D. melanogaster* S2 cell line secreting EgAgB8/1 was generated. To that end, stable S2 cells lines were cultured in Schneider´s *Drosophila* media (Gibco, Thermo Fisher Scientific) containing 10% Foetal Bovine Serum (FBS, Gibco) at 28°C. Cultured S2 cells (1.0 - 2.5 x 10^6^ cells/ml) were transfected with 2 µg of pDroEx-EgAgB8/1 and 0.1 µg of pCoPURO (a puromycin resistant plasmid, Invitrogen) using Effectene Transfection Reagent (Qiagen) and following the manufacturer’s recommendations. The presence of pDroEx-EgAgB8/1 in puromycin-resistant cells was controlled by PCR using the generic primers. During subculture of transfected S2 cells, Schneider´s *Drosophila* medium was progressively substituted by Xpress medium (Lonza, Switzerland) without FBS. When transfected S2 cell culture reached 7 x 10^6^ cell/mL, EgAgB8/1 expression was induced with 5 µM CdCl_2_, and the Xpress medium containing CdCl_2_ was added to replenish nutrients at 3 and 5 days-post-induction (dpi). At the end point, cell suspension was centrifuged and the supernatant was adjusted at pH 8.0 and kept ON at 4°C. Afterwards, the supernatant was clarified by centrifugation (6,000 x *g*, 20 min) followed by filtration at 0.22 µm, and fractionated on a Strep-Tactin XT-agarose column (5 mL, IBA, Life Sciences) using 100 mM Tris pH 8.0, containing 150 mM NaCl, 5 mM EDTA, 20 μM BHT and antibiotic/antimycotic as equilibration buffer and adding 25 mM biotin for elution. The eluted fraction was then applied on the anti-EgAgB clone 1-Sepharose column, previously equilibrated in PBS_EBAb_. After washing, rEgAgB8/1 was eluted using 0.1 M glycine pH 2.5, and afterwards equilibrated and maintained in PBS_EBAb_ as described for immunopurified EgAgB. Induction time for rEgAgB8/1 production was evaluated at 4, 7 and 12 dpi in terms of yield and particle size. Analysis of endotoxin level was carried out by LAL assay (Beltran Zunino, Montevideo).

### 2.11 Native and denaturing gel electrophoresis and western blot analysis

For SDS-PAGE analysis, all samples (HF fractions, nEgAgB and rEgAgB8/1) were resolved on 15% polyacrylamide gels under reducing conditions (40 µM DTT) according to Laemmli (Laemmli, 1970). Gels were stained with Colloidal Coomassie G-250 (Sigma-Aldrich) or transferred onto PVDF membranes (Millipore-Merck, USA) using a Mini Protean Blot Module (Bio-Rad) following the manufacturer’s instructions. Blots were blocked 1 hour at RT in PBS 5% non-fat dry milk, and then incubated with mAb EB7 (diluted hybridoma supernatant) for 1 hour at RT. They were washed with PBS 0.05% Tween-20 and incubated for 1 hour at RT with anti-mouse IgG peroxidase conjugate (diluted 1:4,000; Thermo Scientific). After washing, blots were developed using ECL Supersignal West Pico chemiluminescent substrate (Thermo Scientific) in the G-Box F3 image capture equipment (Syngene).

Both nEgAgB and rEgAgB8/1 were analysed by native gradient gel acrylamide electrophoresis (GGE) using 4-20% gradient gels and the Mini-PROTEAN TGX (Biorad, Life Science, Mexico) and following the instructions of the fabricant. For size comparison the human plasma lipoproteins LDL (density range from 1,019 g/mL - 1,063 g/mL), HDL_2_ (density range from 1,063 g/mL- 1,125 g/mL) and HDL_3_ (density range from 1,125 g/mL- 1,210 g/mL), purified by sequential ultracentrifugation (Havel et al., 1955) were run in parallel. Gels were stained with Coomassie Brilliant Blue or Sudan Black for visualisation of protein and lipid components, respectively. The relative mobility of all samples was estimated using a ChemiDoc 2,000 densitometer and the software Quantity One (Bio-Rad Laboratories SA, Life Science Group).

### 2.12 Lipid analysis

Lipids were analysed by HPLC-MS using a ZICpHILIC column (150 x 4.6 mm, 5 µm particle size) supplied by Hichrom Ltd. (Reading, UK) with a mobile phase consisting of 20 mM ammonium carbonate in HPLC-grade water (solvent A) and acetonitrile (solvent B), at a flow rate of 0.3 mL/min. The elution gradient was an A:B ratio of 20:80 (v:v) at 0 min, 80:20 (v:v) at 30 min, 92:8 (v:v) at 30 min 92:8 (v:v) at 35 min, 20:80 (v:v) at 36 min, and 20:80 (v:v) at 45 min. An ACE C4 column (Hichrom Ltd) was used to estimate the unsaturated FAs. The mobile phase for the elution of the ACE C4 column consisted of 1 mM acetic acid in water (A) and 1 mM acetic acid in acetonitrile (B) at a flow rate of 0.4 mL/min. The elution gradient was 40% B (0 min), 100% B (30-36 min) and 40% B (37-41 min). The HPLC-MS system consisted of a Dionex 3,000 HPLC pump interfaced with Orbitrap Exactive mass spectrometer used in positive/negative switching mode. The instrument was calibrated according to the manufacturer’s instructions and operated at 50,000 resolution. The needle voltage was 4.5 kV in positive mode and 4 kV in negative ion mode, the heated capillary temperature was 320°C and the sheath and auxiliary gases 50 and 17 arbitrary units respectively. The data was acquired using Xcalibur 2.0 software (Thermo Fisher Scientific) and the data sets were extracted and aligned by using m/z Mine 2.14.2 and the data was searched against an in-house database of metabolites. All identified lipids were within 5 ppm of their exact masses. Further processing of the data was carried out by using Metaboanalyst 4.0. For a graphical representation of phospholipid and FA content, heatmaps were built using Graphpad Prism 9.4.1 software.

### 2.13 Dynamic light scattering

The hydrodynamic radius (R_H_) and the polydispersity index (PDI) of EgAgB particles were measured by dynamic light scattering (DLS), using the Zetasizer NanoS (Malvern Panalytical, United Kingdom). Samples (70 µL, 1 mg/mL in PBS_EB_) were placed in disposable cuvettes (UVette, Eppendorf) and pre-incubated at 25°C before measurement. Data from triplicate measurements were averaged and analysed with Zetasizer Software v7.13 (Malvern Panalytical) to obtain size distribution of the samples (weighted by volume as assuming sphericity and homogeneity of particles). Reported R_H_ values were calculated as the mean value for the main peak.

### 2.14 Mice

BALB/c mice (female, 8- to 12-week-old) were acquired from DILAVE (Dirección de Laboratorios Veterinarios, Ministerio de Ganadería, Agricultura y Pesca, Uruguay) or Institut Pasteur Montevideo (IP-Montevideo, Uruguay). Animal manipulation and husbandry were done in accordance with the ethical committee guidelines of the Honorary Commission of Animal Experimentation (CHEA) from UdelaR.

### 2.15 *In vitro* EgAgB effect on macrophage activation

The human monocyte-like cell line THP-1 (American Type Culture Collection, USA) was maintained in RPMI medium (RPMI 1640 culture medium containing 10 mM HEPES, 1.5 g/L sodium bicarbonate, 1 mM sodium pyruvate, 2 mM glutamine, 100 U/mL penicillin, 0.1 mg/m streptomycin and 250 ng/mL amphotericin B) plus 10% (v/v) FBS. Cells were maintained at 37°C in a humidified atmosphere with 5% (v/v) CO_2_ and sub-cultured every 3-4 days to maintain cell density between 0.2 and 1.0×10^6^ cells/mL Macrophage differentiation was induced by stimulation of THP-1 cells (0.2x10^6^/well in 96-well tissue culture plates) with PMA (50 ng/mL, Sigma-Aldrich) in RPMI supplemented with 5% FBS for 48 hours. Afterwards, cells were cultured in a medium containing 5% FBS for additional 24 hours.

Murine bone marrow-derived macrophages (BMDM) were prepared following a procedure approved by CHEA (protocol N° 538, Exp. No. 101900-000999-17). To that end, bone marrow precursors from Balb/C mice (IP-Montevideo) were differentiated in the presence of conditioned medium from the L929 cell line, containing M-CSF, for 7 days as described in (Mylonas et al., 2013). Cells were then plated (0.5x10^6^ cell/well in 48-well culture plates) using DMEM containing FBS 1% (v/v), and cultured ON at 37°C in a humidified atmosphere with 5% (v/v) CO_2_.

THP-1 macrophages or BMDM were exposed to nEgAgB or rEgAgB8/1 (at 1 or 10 µg/mL in analytical triplicates) in the presence or absence of LPS (10 ng/mL, *E. coli* O127:B8, Sigma-Aldrich), using PBS_EBAb_ (vehicle) as control. Cell viability and responses (cytokines and nitrite in supernatants or expression of cell surface molecules) were assessed 24 hours post-stimulation using the methods described below.

### 2.16 *In vivo* EgAgB effect on mouse peritoneal cavity

Animal manipulation and procedures (protocol N° 542, Exp. N° 101900-000972-17) were approved by CHEA. Balb/C mice (DILAVE) were randomly distributed in four groups (n=5 each) and intraperitoneally (i.p.) injected as follows: Vehicle group: PBS_EBAb_ (EgAgB vehicle) followed by sterile saline solution (SS, as LPS vehicle); EgAgB groups: 50 μg of nEgAgB or rEgAgB8/1 in PBS_EBAb_ followed by SS; LPS group: PBS_EBAb_ followed by 15 μg of LPS in SS (*E. coli* O127:B8, Sigma-Aldrich); EgAgB+LPS groups: 50 μg of nEgAgB or rEgAgB8/1 in PBS_EBAb_ followed by 15 μg of LPS in SS. To avoid putative interactions before administration, EgAgB was injected 10 min before LPS. After 4 or 24 hours-post-injection (hpi), mice were euthanized and peritoneal cells were collected by lavage with RPMI containing 0.2% FBS and 2 mM EDTA and analysed by flow cytometry as described below. In addition, peritoneal content was retrieved by peritoneal lavage with 1 mL of 0.2% (v/v) FBS in RPMI and maintained at -80°C until cytokine analysis by ELISA.

### 2.17 Measurement of cell responses

Cell viability was assessed on the basis of the mitochondrial reductase activity, which convert the water-soluble yellow dye 3-(4,5-dimethylthiazol-2-yl)-2,5-diphenyltetrazolium bromide (MTT, Bio Basic Inc., Canada) to the insoluble, chromogenic, formazan (Kumar et al., 2018). Briefly, macrophage cultures were cultured in the presence of MTT (200 μg/mL in PBS containing 0.1% glucose) for 3 hours at 37°C and 5% CO_2_. After washing with PBS, DMSO was added and the produced formazan was determined by measuring the absorbance at 560 nm.

Cytokine responses. IL-1β, IL-6, IL-12p40, IL-10 and interferon-β (IFN-β) were measured in culture supernatants by capture enzyme-linked immunosorbent assay (ELISA) employing OptEIA kits (BD Biosciences), according to the manufacturer’s instructions.

Nitric oxide (NO·) response. Nitrite levels were determined by the colorimetric Griess assay as an indicator of NO· generation. Briefly, macrophage culture supernatants were transferred (50 μL/well) to 96-well flat-bottom plates, and then 50 μL of sulfanilamide (Sigma, 1% wt/vol in 2.5% H_3_PO_4_) and 50 μL of naphthylethylenediamine dihydrochloride (Sigma, 0.1% wt/vol in 2.5% H_3_PO_4_) were added to the supernatants. After 5 min incubation, the absorbance at 540 nm (A_540_) was measured and converted to nitrite concentration based on a NaNO_2_ standard curve.

Cellular characterization by flow cytometry. Cells (between 0.4-0.5x10^6^) were incubated at RT for 10 min with Live/dead-Aqua viability probe (1:500 (v:v) dilution, Thermo Fisher Scientific) and then for 20 min at RT with 15% normal rat serum in buffer PBS containing 0.1% (w/v) BSA, 2 mM EDTA, pH 7.1 (FACS). Then, cells were incubated on ice for 30 min with antibodies to surface molecules associated with cell activation CD86, CD40, and MHC-II. In the case of peritoneal cells, antibodies to the phenotypic markers F4/80, CD19, and Ly6C were also included (**Supplementary Table S1**). Afterwards, cells were washed with FACS buffer. Data were acquired on Facs Canto II cytometer and analysed using the FlowJo package. The gating strategy is shown in **Supplementary Figure 3**. Resident large peritoneal macrophages (LPM) were defined as CD19^−^F4/80^++^Ly6C^-^ while small peritoneal macrophages (SPM) as CD19^−^F4/80^+/-^SSC^low^Ly6C^-^MHC-II^++^cells. Infiltrating monocytes were identified as CD19^−^F4/80^+/-^SSC^low^Ly6C^++^MHC-II^-^. When quantifying cell surface molecule expression, the fluorescence intensity (FI) corresponding to the FMO (fluorescence minus one) control was subtracted. In addition, for comparison between mice groups, FI values were normalised to the control (vehicle treated mice).

### 2.18 Data analysis

The number of independent experiments and internal repetitions used for statistical analysis and summarised in the graphs shown is given in each figure legend. For graphical presentation purposes, some data were normalised to the control group or to the LPS response (as indicated); however, statistical analyses were always carried out on the crude data. Significances are indicated by asterisks in figures and are explained in the figure legends. In the case of *in vitro* studies, multiple comparison was performed by two-way ANOVA with Tukey’s *post hoc* test, using Graphpad Prism 9.4.1. In the case of *in vivo* studies, data were analysed by a nonparametric method, since they did not reach normality and homogeneity of variances. Specifically, the Mack Skillings exact test for a two-way layout was applied (Hollander et al., 2015). When the MackSkillings test resulted in a statistic with a *p*-value of less than 0.05, the test was followed by the *post hoc* multiple-comparison test described by Conover (Conover, 1999) and the Benjamini and Hochberg correction (McDonald, 2009). Throughout the paper, the symbols *, **, and *** represent *p*-values of less than 0.05, 0.01, and 0.001, respectively.

## 3 Results

### 3.1 Development of a VHH specific for EgAgB8/1

**Figure 1:**
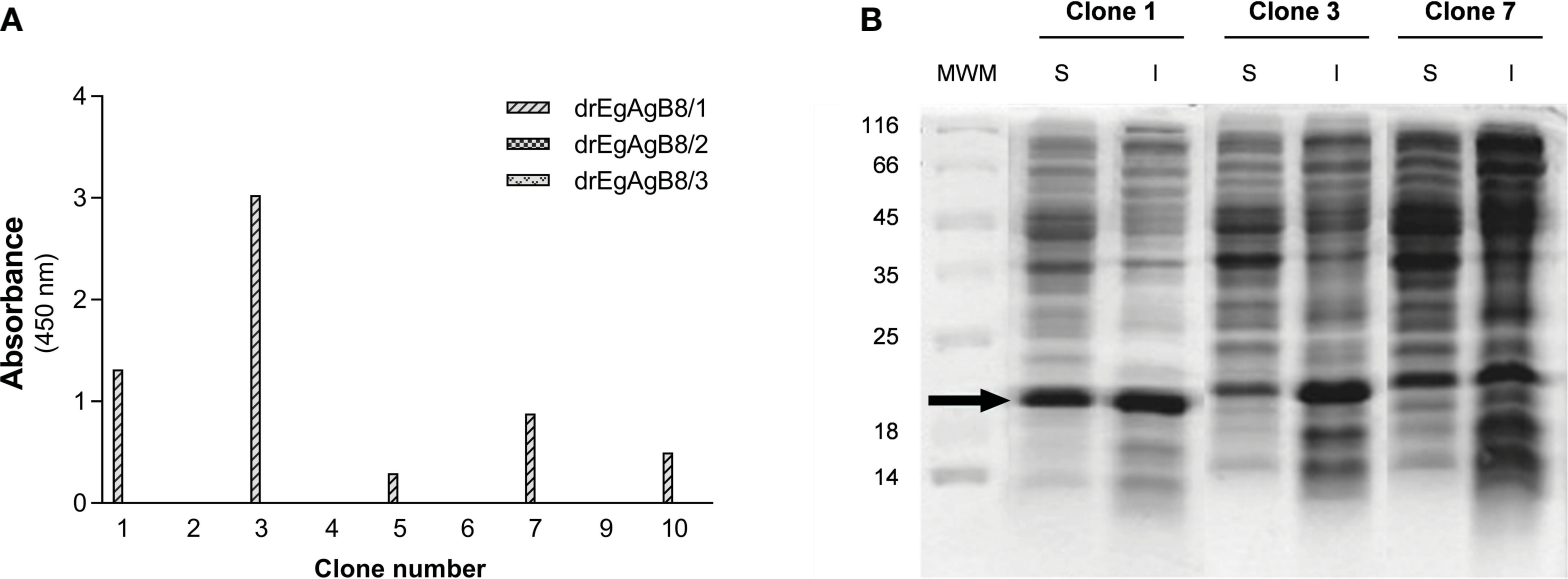
Characterization of anti-EgAgB VHHs/VHs clones: binding to different EgAgB8 recombinant subunits and soluble expression. **(A)** The supernatants of anti-EgAgB VHH/VHs clones were assessed for binding to ELISA wells coated with drEgAgB8/1-3 (0.5 µg/well). Note that no reaction was detected for drEgAgB8/2 and drEgAgB8/3. **(B)** SDS-PAGE analysis of soluble (S) and insoluble (I) fractions of clones 1, 3 and 7. Samples were run on a 12.5% polyacrylamide gel and protein bands were stained using Coomassie Blue staining.

A VHHs/VHs phage display library, cloned in the pCOMB3X phagemid vector, was prepared using RNA extracted from peripheral mononuclear cells of a llama immunised with EgAgB (Ld_f_). After performing two rounds of panning, screening of EgAgB-specific clones was performed by ELISA using Ld_f_ for coating. All (10) individual clones expressing VHHs/VHs in the second output of the panning were positive for Ld_f_-coated wells, and none of them showed reactivity against BSA-coated wells, indicating they were specific for Ld_f_ (**Supplementary Figure S4**). The full coding sequences for VHHs/VHs genes of the second output were then transferred en masse from the pCOMB3x to the pINQ-HAH6 vector, to improve the VHH/VH expression efficacy. The transference of the VHHs/VHs genes was evaluated by performing the same ELISA previously done for the screening, finding that nine out of ten original clones were positive for Ld_f_ (**Supplementary Figure S5**). Primary sequencing of the nine Ld_f_ positive clones allowed identifying five distinct sequences (**Supplementary Figure S6**) that exhibited a VHH-specific amino acid pattern in framework 2, thereby confirming that all isolated clones were VHHs. The subsequent characterization of their binding specificity was carried out by ELISA using the delipidated EgAgB8 subunits expressed in *E. coli* (drEgAgB8/1-3), showing that five out of nine positive clones recognized drEgAgB8/1 and none of them were positive for drEgAgB8/2 nor drEgAgB8/3 (**Figure 1A**). The predominance of VHH positive clones specific for EgAgB8/1 agrees with its higher abundance in the nEgAgB present in bovine HF compared to the EgAgB8/2-5 subunits (Folle et al., 2017). Since anti-EgAgB VHHs were developed to allow affinity purification of nEgAgB and its recombinant form, we chose the EgAgB-specific clone that produced higher amounts of soluble VHH as selection criteria. Clones 1, 3 and 7, showing the highest signals for EgAg8/1 by ELISA (**Figure 1A**), were grown and the VHH expression was induced with IPTG. According to SDS-PAGE of soluble and insoluble fractions obtained from cell lysates, clone 1 yielded the highest amount of soluble VHH (**Figure 1B**). This clone was further expressed, purified and immobilised on CNBr-Sepharose for immunoaffinity chromatography.

### 3.2 Immunoaffinity with anti-EgAgB clone 1 allowed obtaining nEgAgB and rEgAgB8/1 preparations suitable for macrophage functional studies

**Figure 2:**
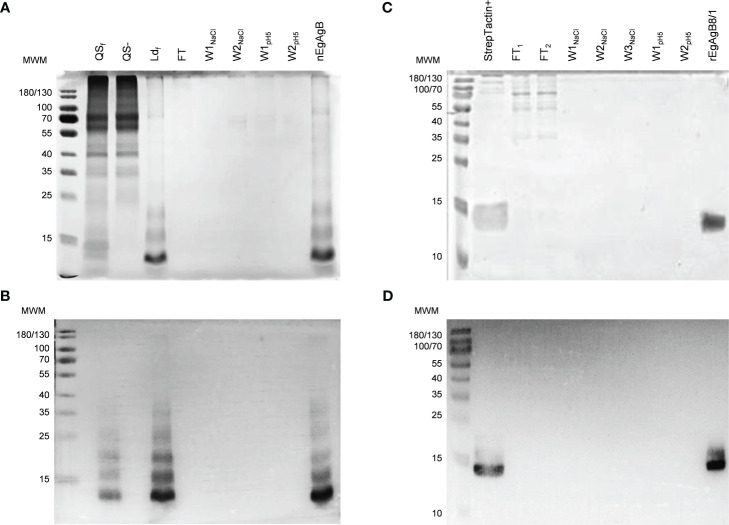
Immunoaffinity yielded pure nEgAgB and rEgAgB8/1 preparations. Analysis by SDS-PAGE **(A)** and Western Blot **(B)** of the fractions corresponding to the purification protocol of nEgAgB from HF: bound (QS+) and unbound (QS-) fractions to Q-Sepharose, the low-density fraction (Ld_f_) obtained by ultracentrifugation of QS+ on a KBr density gradient, and the fractions obtained by immunoaffinity of Ld_f_ on anti-EgAgB clone1–Sepharose. Notice the typical EgAgB pattern with regularly spaced bands. Analysis by SDS-PAGE **(C)** and Western Blot **(D)** of the fractions corresponding to the purification protocol of rEgAgB8/1 from the supernatant collected at 7 dpi: the fraction bound to Strep-Tactin XT-agarose column (StrepTactin+) and those obtained by its subsequent fractionation on anti-EgAgB clone1–Sepharose. In all cases samples were run on a 15% polyacrylamide gel under reducing conditions (6 mM DTT) and protein bands were detected by Colloidal Coomassie Blue staining. Western blot was performed using the mAb EB7. FT, flow through; W_NaCl_, washes with 1M NaCl; W_pH5_, washes using a pH5.

The native form of EgAgB was purified to homogeneity by immunoaffinity of Ld_f_ using anti-EgAgB clone1-Sepharose. The unbound proteins were washed with a high salt concentration (1M) and by decreasing pH, and as expected, the nEgAgB was eluted at pH 2.5 as a yellowish fraction. SDS-PAGE and Western blot analysis showed that the fraction eluted at pH 2.5 contained high-purity nEgAgB (**Figures 2A, B**, respectively), exhibiting the typical pattern with regularly spaced bands in both analyses. This pattern was almost identical between Ld_f_ and nEgAgB, indicating no detectable differences in protein composition. However, analysis by the LAL assay revealed higher endotoxin levels in Ld_f_ (≥ 0.9 ng/µg protein) than nEgAgB (≤ 0.02 ng/µg protein), allowing to test nEgAgB but not Ld_f_ at 10 µg/mL concentrations in studies for examining macrophage activation. These results were reproduced in six nEgAgB batches derived from different HF pools, suggesting that immunoaffinity contributes to obtaining high-quality nEgAgB preparations suitable for macrophage studies. Only 2.6 mg (protein) of Ld_f_, containing approximately 0.0114 micromoles of nEgAgB, saturated the anti-EgAgB clone 1-Sepharose column; this amount was low compared to the estimated 0.75 micromoles (15 mg) of anti-EgAgB VHH immobilised in the Sepharose matrix. Assuming a 1:1 binding ratio, the recovered nEgAgB represented only 1.5% of the theoretical yield, estimated if this VHH would be in a solution.

Expression of rEgAgB8/1 in the S2 cell line was carried out by transfection with the pDroEx-EgAgB8/1 expression vector (containing the sequence EGR_06805) and pCoPURO to select transfected cells using puromycin. The rEgAgB8/1 was detected in cell culture supernatants at 4 dpi by SDS-PAGE and Western blot, and purified using Strep-Tactin XT-agarose column after 4, 7 or 12 dpi (see **Supplementary Figures S7A, B**) followed by immunoaffinity with the anti-EgAgB clone1-Sepharose using the same procedure as that for purifying nEgAgB. The SDS-PAGE and Western blot analyses showed that rEgAgB8/1 was pure and had a lower degree of oligomerisation than nEgAgB (**Figures 2C, D**). Using a 50 mL culture, lower amounts of rEgAgB8/1 were obtained at 4 dpi (48 µg) compared to 7 and 12 dpi (117 and 129 µg, respectively). Transfected S2 cells showed a high viability within the first week of cell culture, but it decreased progressively from one week on. These results, together with the characterisation of rEgAgB described below, led us to select 7 dpi as the optimal induction time for expression. At this condition, rEgAgB8/1 preparation showed a modest but reproducible yield in six independent batches (about 3 mg per litre of culture medium). Similarly to nEgAgB, the endotoxin levels detected in rEgAgB8/1 preparations (≤ 0.02 ng/µg protein) enable its use at up to 10 µg/mL in macrophage functional studies.

### 3.3 nEgAgB and rEgAgB8/1 exhibited differences in size and lipid composition

**Figure 3:**
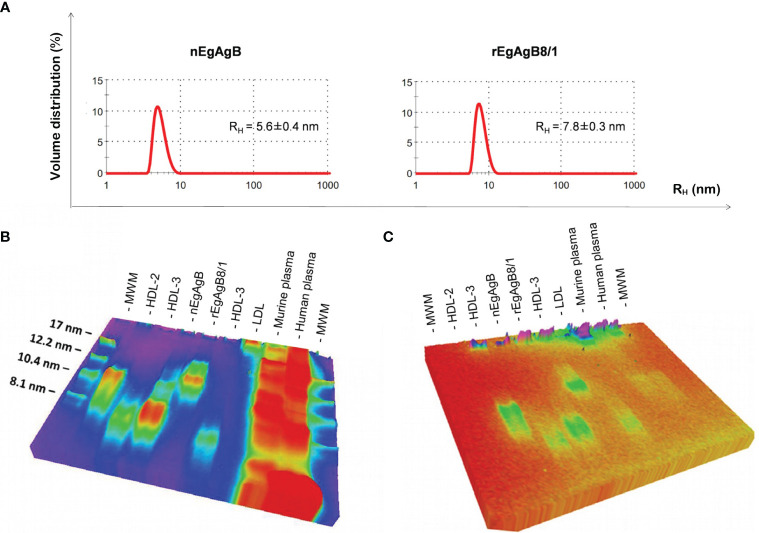
rEgAgB8/1 is a complex lipoprotein slightly higher than nEgAgB in size. **(A)** Determination of the hydrodynamic radius (R_H_) by DLS. Samples were analysed in triplicates and results are shown as the volume distribution (%) plotted against the hydrodynamic radius (R_H_). Results are representative of four and two independent batches of nEgAgB and rEgAgB8/1, respectively. Both samples showed a monomodal size distribution of particles. The mean R_H_ value obtained is indicated. **(B, C)** Analysis of the protein and lipid content by native gel electrophoresis, using Coomassie Brilliant Blue or Sudan Black for visualization, respectively. nEgAgB and rEgAgB8/1 were analysed in parallel to HDL_2_, HDL_3_, LDL and murine and human plasma samples. High amounts of plasma samples were analysed in order to detect lipid components. Results showed that nEgAgB and rEgAgB8/1 are complex lipoproteins, with a principal component of around 7 and 12 nm in diameter, respectively. MW, Molecular weight standard.

The analysis by DLS of immunopurified nEgAgB particles showed a multimodal size distribution (weighted by intensity) corresponding to a principal peak (weighted by volume) with an average hydrodynamic radius (R_H_ ± SD) of 5.6 ± 0.4 (**Figure 3A**) and overall PDI values ranging between 0.236 to 0.419, indicating moderate to broad overall polydispersity. The rEgAgB8/1 preparations obtained at 4, 7 and 12 dpi were also polydisperse (PDI between 0.256 to 0.443) and had a monomodal size distribution with R_H_ ± SD of 8.3 ± 0.5, 7.8 ± 0.3 and 13.8 ± 0.7, respectively (**Supplementary Figure 7C**). This revealed that after 7 dpi the particle size of rEgAgB8/1 was about 2-fold larger than nEgAgB. In this scenario, we only used for biochemical characterization and functional assays rEgAgB prepared from cell culture supernatants obtained at 7 dpi.

**Figure 4:**
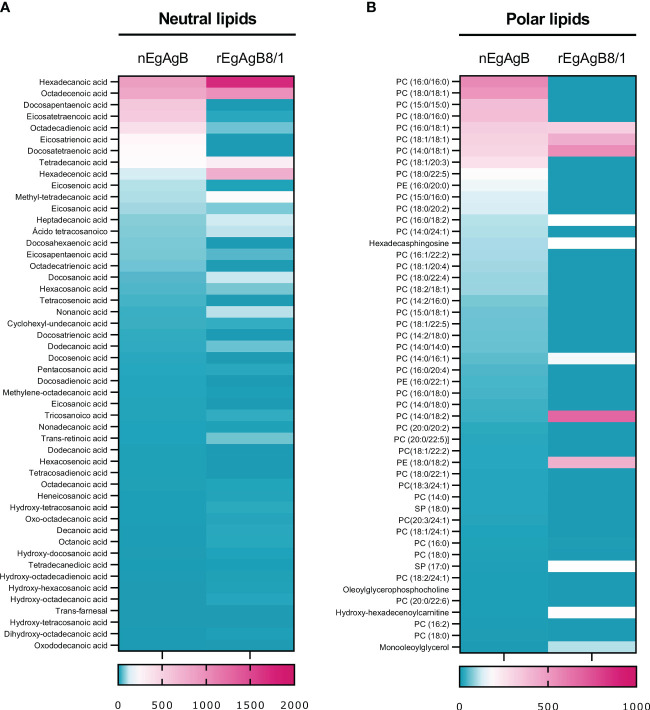
The content of neutral and polar lipids of rEgAgB8/1 showed differences with that of nEgAgB. A quantitative lipid analysis was performed by HPLC-MS employing ACE C4 and ZICpHILIC columns for characterising the FAs and polar lipids, respectively. The 50 most abundant **(A)** neutral and **(B)** polar lipids are represented as heatmaps. For each heatmap, the relative content of each lipid species was normalised by the total amount of detected lipids. Data correspond to the analysis of nEgAgB prepared from seven hydatids and rEgAgB8/1 prepared from two independent productions, and are expressed as the mean of technical triplicates for neutral and duplicates for polar lipids.

Protein and lipid analysis by native (non-denaturing) gel electrophoresis showed that both nEgAgB and rEgAgB8/1 displayed characteristics of complex lipoproteins, with a principal component of 7 and 12 nm in diameter, respectively (**Figures 3B, C**). The sizes of the nEgAgB and rEgAgB8/1 were similar to those shown by the plasma fraction of human HDL_3_ and HDL_2_, respectively. These results suggest that the rEgAgB8/1 subunit could also undergo oligomerization during lipid acquisition. Analysis by HPLC-MS of the lipid composition was focused on the phospholipid species and total FAs, which were expected to be in tight association with EgAgB subunits at the lipoprotein surface, conditioning the interactions established with putative cell receptors (Obal et al., 2012). Both EgAgB preparations revealed differences in their 50 most abundant FAs and phospholipids bound to the protein moiety. In agreement with previous studies, C16:0, C18:1, C22:5, C20:4, and C18:2 were the predominant FAs detected in nEgAgB (Obal et al., 2012), while C16:0 showed a high abundance in rEgAgB8/1 followed by C18:1 and C16:1 and C14:0 (**Figure 4**). In addition, nEgAgB contained a wider variety of phospholipids than rEgAgB8/1, with phosphatidylcholine species (with different FAs occupying positions 1 and 2 in the glycerol) being predominant in both (**Figure 4**).

### 3.4 Both nEgAgB and rEgAgB8/1 interfered with LPS-driven macrophage activation *in vitro*

**Figure 5:**
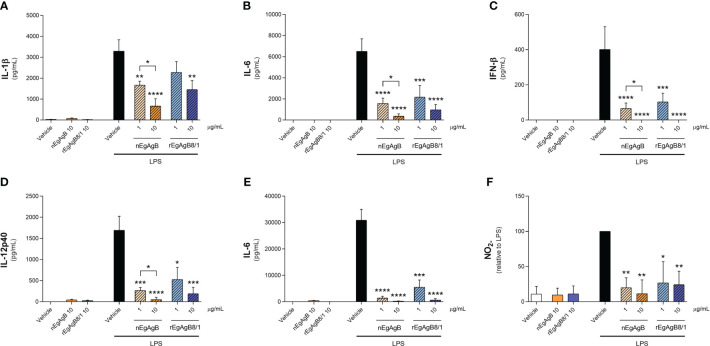
nEgAgB and rEgAgB8/1 interfered with the LPS-induced production of cytokines and nitrite by macrophages. THP-1 and BMDM macrophages were stimulated with nEgAgB, rEgAgB8/1 or vehicle (PBS_EBAb_) in the absence or presence of LPS. After stimulation for 24 hours cytokine secretion and nitrite (indicative of ·NO production) levels were determined in cell supernatants. The levels of **(A)** IL-1β, **(B)** IL-6, **(C)** IFN-β for THP-1 macrophages, and **(D)** IL-12p40, **(E)** IL-6 and **(F)** nitrite for BMDM are plotted as the mean ± SEM of three independent experiments with analytical triplicates. Data showing significant differences from LPS are indicated with * (two-way ANOVA and Tukey’s test, *p < 0.05, **p < 0.01, ***p < 0.001, ****p < 0.0001).

**Figure 6:**
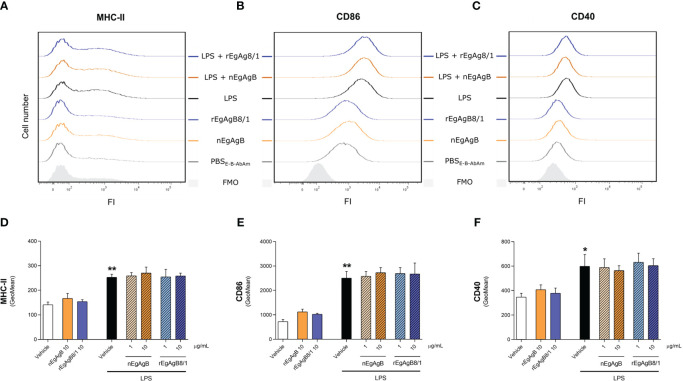
nEgAgB and rEgAgB8/1 did not alter CD40, CD86 or MHC-II expression in BMDM, in the presence or absence of LPS. BMDM were stimulated with nEgAgB, rEgAgB8/1 or vehicle (PBS_EBAb_) in the absence or presence of LPS. After 24 hours, MHC-II, CD86 and CD40 expression was measured by flow cytometry. **(A-C)** Representative histograms of the fluorescence intensity (FI) obtained for MHC-II, CD86 and CD40. **(D-F)** Bar graphs represent MHC-II, CD86 and CD40 surface expression (mean FI ± SEM) corresponding to two independent experiments with analytical triplicates (two-way ANOVA and test of Tukey, *p < 0.05, **p < 0.01).

Immunopurified EgAgB preparations did not alter macrophage viability at the assayed concentrations (up to 20 µg/mL, **Supplementary Figure S8**). At 10 µg/mL, both nEgAgB and rEgAgB8/1 did not induce *per se* the secretion of the pro-inflammatory cytokines IL-1β and IL-6 on THP-1 macrophages (**Figures 5A, B**, respectively), but at 20 µg/mL triggered the production of low levels of IL-1β (**Supplementary Figures S9A, B**, respectively). These results contrast with Ld_f_ effects on THP-1 macrophages (**Supplementary Figures S9A, B**) and agree with previous results achieved with nEgAgB immunopurified using the EB7 monoclonal antibody [9]. Similar results were obtained using BMDM, suggesting that concentrations up to 10 µg/mL of both nEgAgB and rEgAgB8/1 did not induce *per se* significant pro-inflammatory effects and thus, were adequate for examining immunomodulation properties. On the other hand, 1 µg/mL of nEgAgB was high enough to inhibit the cytokine secretion induced by LPS in cultured THP-1 macrophages and BMDM. We observed a dose-dependent reduction of IL-1β, IL-6 and IFN-β secretion in activated THP-1 macrophages (**Figures 5A-C**) and of IL-6 and IL-12p40 in activated BMDM (**Figures 5D, E**). It is worth mentioning that in the assayed conditions, IL-1β and IFN-β secretion by BMDM were undetectable in control and LPS-stimulated macrophages. rEgAgB8/1 behaved similarly to nEgAgB, interfering with LPS-mediated responses in THP-1 macrophages and BMDM (**Figures 5A-E**). However, rEgAgB8/1 appeared to exhibit a lower ability to inhibit IL-1β secretion than the native lipoprotein (**Figure 5A**) since inhibition was achieved using 10 but not 1 µg/mL. The EgAgB effects on LPS-induced ·NO response were analysed only in BMDM because nitrite levels were not detectable in LPS-stimulated THP-1 macrophages. Results showed that nEgAgB and rEgAgB8/1 did not induce ·NO response *per se* and caused a decrease in LPS-induced ·NO generation, even when tested at 1 µg/mL (**Figure 5F**). Finally, EgAgB’s ability to modulate the LPS-induced expression of cell surface MHC-II and the costimulators CD40 and CD86 in BMDM was examined by flow cytometry (**Figure 6**). The basal cell surface expression of these markers on control macrophages (vehicle-treated) was not affected by nEgAgB and rEgAgB8/1, showing at best a marginal elevation. In contrast, LPS caused a consistent, although modest, significant increase in the relative abundance of cell surface MHC-II (1.8-fold), CD86 (3.4-fold) and CD40 (1.7-fold). Neither nEgAgB nor rEgAgB8/1 controlled the LPS-induced upregulation of these macrophage markers at the cell surface.

### 3.5 Compared to nEgAgB, rEgAgB8/1 showed a modest modulation of LPS-induced cytokine secretion and macrophage activation in the peritoneum

**Figure 7:**
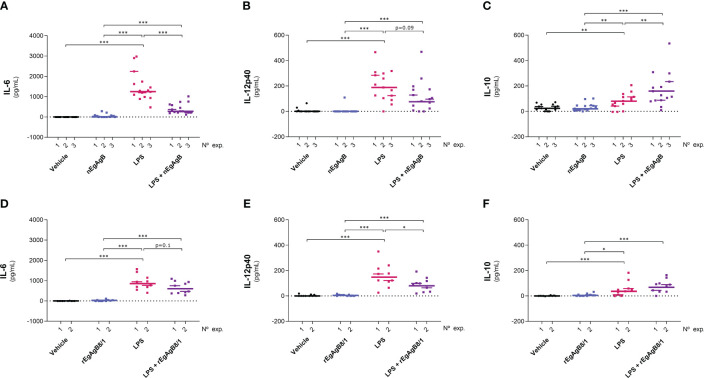
nEgAgB and rEgAgB8/1 altered the cytokine production induced by LPS in the peritoneal cavity. nEgAgB or rEgAgB8/1 (50 µg/mouse) and PBS_EBAb_ (vehicle control) were i.p. injected in Balb/c mice in the absence or presence of LPS (15 µg/mouse). Graphs show the peritoneal levels of IL-6 **(A, D)**, IL-12p40 **(B, E)** and IL-10 **(C, F)** after 4 hpi for 3 and 2 independent experiments with nEgAgB and rEgAgB8/1, respectively. Each point in the graphs represents an individual (n=5 per group). The median value for each independent experiment is shown as a thin and short horizontal line while the median value corresponding to the set of independent experiments is shown as a thick and long horizontal line. The asterisks denote differences between groups (Mack Skillings two-way non-parametric exact test, followed by the Conover *post hoc* multiple-comparison test with Benjamini and Hochberg correction (*p < 0.05, **p < 0.01, ***p < 0.001).

The EgAgB effects on monocyte and macrophage activation were examined *in vivo* using an inflammatory model based on the injection of LPS in the mouse peritoneal cavity. In this model, 15 µg of LPS generated a moderate response, characterised by the presence in the peritoneal lavage of IL-6, IL-12p40 and variable amounts of IL-10 (undetectable in some experiments) at 4 hpi. None of these cytokines was detected at 24 hpi. Following our *in vitro* observations, nEgAgB modulated the LPS-induced cytokine responses by significantly decreasing IL-6, though marginally, also IL-12p40 (**Figures 7A, B**, respectively), and by increasing IL-10 levels (**Figure 7C**). rEgAgB8/1 caused similar modulatory effects despite these trends only reaching statistical significance for IL-12p40 reduction (**Figures 7D-F**).

**Figure 8:**
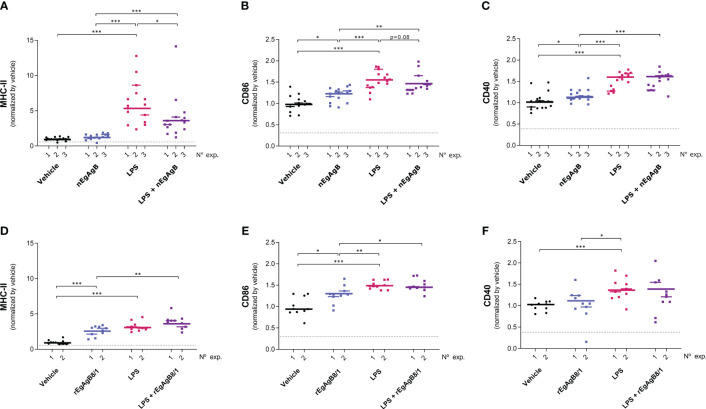
nEgAgB modulated the LPS-induced surface expression of MHC-II, but not of CD86 and CD40 in LPM; rEgAgB8/1 did not provoke alterations of any of these surface molecules. nEgAgB or rEgAgB8/1 (50 µg/mouse) and PBS_EBAb_ (vehicle control) were i.p. injected in Balb/c mice in the absence or presence of LPS (15 µg/mouse). Peritoneal cells were collected after 24 hpi and analysed by flow cytometry. Graphs show the surface expression of MHC-II **(A, D)**, CD86 **(B, E)** and CD40 **(C, F)** in LPM (defined as CD19-Ly6C-F4/80++ cells) corresponding to 3 and 2 independent experiments using nEgAgB and rEgAgB8/1, respectively. Data is presented as the FI normalised to the vehicle control. Each point in the graphs represents an individual (n=5 per group). The median value for each independent experiment is shown as a thin and short horizontal line while the median value corresponding to the set of independent experiments is shown as a thick and long horizontal line. The asterisks denote differences between groups (Mack Skillings two-way non-parametric exact test, followed by the Conover *post hoc* multiple-comparison test with Benjamini and Hochberg correction (*p < 0.05, **p < 0.01, ***p < 0.001).

**Figure 9:**
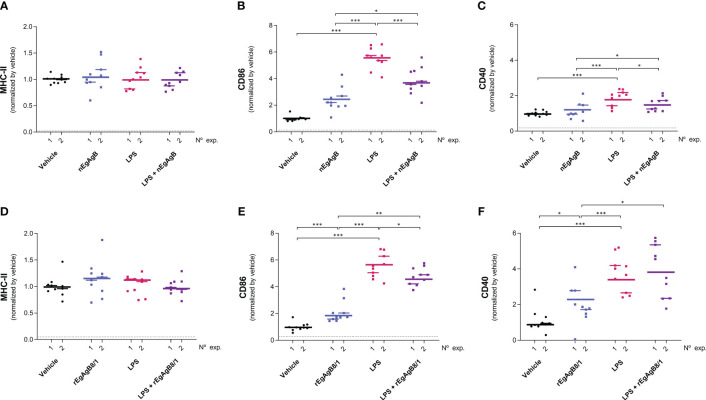
nEgAgB modulated the early induction of CD86 and CD40 promoted by LPS in SPM while rEgAgB8/1 only affected CD86 expression. nEgAgB or rEgAgB8/1 (50 µg/mouse) and PBS_EBAb_ (vehicle control) were i.p. injected in Balb/c mice in the absence or presence of LPS (15 µg/mouse). Peritoneal cells were collected after 4 hpi and analysed by flow cytometry. Graphs show the surface expression of MHC-II **(A, D)**, CD86 **(B, E)** and CD40 **(C, F)** in SPM (defined as (CD19^−^F4/80^+/-^SSC^low^Ly6C^-^MHC-II^++^ cells) corresponding to 3 and 2 independent experiments using nEgAgB and rEgAgB8/1, respectively. Data is presented as the FI normalised to the vehicle control. Each point in the graphs represents an individual (n=5 per group). The median value for each independent experiment is shown as a thin and short horizontal line while the median value corresponding to the set of independent experiments is shown as a thick and long horizontal line. The asterisks denote differences between groups (Mack Skillings two-way non-parametric exact test, followed by the Conover *post hoc* multiple-comparison test with Benjamini and Hochberg correction (*p < 0.05, **p < 0.01, ***p < 0.001).

**Figure 10:**
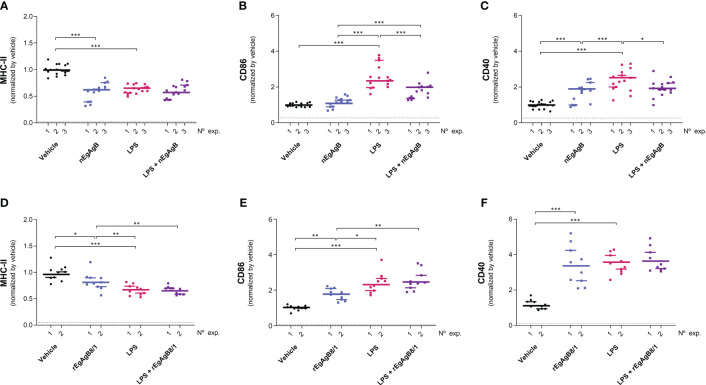
nEgAgB effects on LPS-induced CD86 and CD40 expression in SPM were sustained after 24 hpi. nEgAgB or rEgAgB8/1 (50 µg/mouse) and PBS_EBAb_ (vehicle control) were i.p. injected in Balb/c mice in the absence or presence of LPS (15 µg/mouse). Peritoneal cells were collected after 24 hpi and analysed by flow cytometry. Graphs show the surface expression of MHC-II **(A, D)**, CD86 **(B, E)** and CD40 **(C, F)** in SPM (defined as (CD19^−^F4/80^+/-^SSC^low^Ly6C^-^MHC-II^++^ cells) corresponding to 3 and 2 independent experiments using nEgAgB and rEgAgB8/1, respectively. Data is presented as the FI normalised to the vehicle control. Each point in the graphs represents an individual (n=5 per group). The median value for each independent experiment is shown as a thin and short horizontal line while the median value corresponding to the set of independent experiments is shown as a thick and long horizontal line. The asterisks denote differences between groups (Mack Skillings two-way non-parametric exact test, followed by the Conover *post hoc* multiple-comparison test with Benjamini and Hochberg correction (*p < 0.05, **p < 0.01, ***p < 0.001).

On the other hand, LPS did not modify the total number of peritoneal cells at 4 hpi but caused a slight reduction in this number at 24 hpi (**Supplementary Figure S10**); this is likely due to a significant macrophage migration to the omentum (Cassado et al., 2011) that appears not to be compensated by the recruitment of inflammatory blood cells at this time point. LPS activation of resident and self-renewing peritoneal macrophages, referred to as LPM (CD19^−^F4/80^++^Ly6C^-^), was null in terms of the cell surface expression of co-stimulators CD86 and CD40 at 4 hpi. However, at 24 hpi LPS caused upregulation of cell surface MHC-II and CD86, and a modest increase in CD40 compared to the vehicle (**Figure 8**, **Supplementary Figure S11**). The presence of nEgAgB only partially reduced the LPS-induced increase of cell surface MHC-II in LPM (**Figure 8A**); however, this effect was not reproduced by rEgAgB8/1 (**Figure 8D**). On the other hand, at 4 hpi LPS caused increments of cell surface CD86 and CD40 in the population of small resident peritoneal macrophages derived from blood monocytes, known as SPM (CD19^−^F4/80^+/-^SSC^low^Ly6C^-^MHC-II^++^, **Supplementary Figure S11**). These increments were inhibited in the presence of nEgAgB (**Figures 9A, B**). rEbAgB8/1 mimicked the nEgAgB modulatory effect on CD86 but not on CD40 (**Figures 9C, D**). Similarly, at 24 hpi, LPS promoted cell surface CD86 and CD40 increases while diminished MHC-II in SPM (**Figure 10**). In this case, nEgAgB, but not rEgAgB8/1, inhibited partially LPS-induced increases in CD86 and CD40. LPS-recruited monocytes (CD19^−^F4/80^+/-^SSC^low^Ly6C^++^MHC-II^-^) were not detected until 24 hpi. In the presence of EgAgB preparations, trends to lower expression of CD86 and CD40 in recruited monocytes were observed, but the low number (between 100 and 550) of recruited cells makes the comparison unreliable (**Supplementary Figure S12**). The modulatory effects of EgAgB (both native and recombinant) on LPM and SPM activation by LPS were mostly partial in agreement with the fact that even in the presence of EgAgB, LPS caused some increases in MHC-II and/or cell surface costimulators CD86 and CD40 receptors (indicated as statistically significant differences between EgAgB and EgAgB plus LPS groups, **Figures 8**-**10**). nEgAgB and rEgAgB8/1 caused *per se* a variable activation of LPM and SPM, according to CD86 and/or CD40 increases (**Figures 8**-**10**), which appear to be of lower magnitude than those caused by LPS, except for the CD40 induction by rEgAgB8/1 in SPM at 24 hpi.

## 4 Discussion

Analysis of EgAgB modulatory effects on the activation of innate immune cells requires preserving the native structure of this complex lipoprotein because, up to now, it is uncertain whether EgAgB lipids and/or protein subunits are responsible for these effects. Obtaining nEgAgB confronts the problem of the low EgAgB concentration in HF (< 10 µg/mL, according to purification protocols using bovine fertile HF) together with difficulties in getting parasite material of good quality (non-degenerative, active and fertile hydatids). Efforts made in this work sought to optimise methodological protocols to isolate nEgAgB from HF in amounts and purity appropriate for immunological studies. The VHH anti-EgAgB8/1 clone 1 development contributed to obtaining, by immunoaffinity chromatography, highly pure nEgAgB and rEgAgB8/1. Although this VHH effectively functioned as a specific reagent, its covalent binding to the beads likely interfered with its antigen recognition activity because of the poor yield of the immunoaffinity. Improve the VHH availability for EgAgB binding might be achieved by coupling anti-EgAgB8/1 clone 1 to a Lys-rich polypeptide linker, which provides free NH2 groups for covalent binding to the matrix. Remarkably, the VHH anti-EgAgB8/1 clone 1 would be feasible to produce as a recombinant protein or by chemical synthesis from the known amino acid sequence (Hartmann et al., 2019). Besides, this VHH might be of usefulness for studying EgAgB biochemical properties.

This work provides novel data on EgAgB biochemical and immunomodulatory properties, based on *in vitro* and *in vivo* assays using the native lipoprotein and the recombinant form of EgAgB8/1 expressed in *D. melanogaster* cells. In agreement with previous reports on recombinant EgAgB subunits expressed in *E. coli* (Silva-Álvarez et al., 2015b), rEgAgB8/1 was assembled as a complex lipoprotein. However, compared to nEgAgB, rEgAgB8/1 showed differences in size and lipid composition, having a larger size and less diversity of FAs and phospholipids. The carried lipids likely influence the lipoprotein assembly process and, in turn, could contribute to determine the final particle size. nEgAgB and rEgAgB8/1 probably have similar ability to bind lipids because EgAgB8/1 is by far the most abundant subunit in nEgAgB, and no differences in lipid binding activity were observed between subunits belonging to the two EgAgB subfamilies (Silva-Álvarez et al., 2015b). Therefore, differences in lipid composition between nEgAgB and rEgAgB8/1 likely derive from the pool of lipids available during EgAgB synthesis, secretion and, in the case of the native antigen, putative extracellular remodelling. The higher diversity of FAs found in nEgAgB, including PUFAs having long acyl chains (C20:4, C20:5 and C22:6), confirms our previous results (Obal et al., 2012), demonstrating that EgAgB incorporates and transports essential lipids of host origin (Tielens et al., 2006; Tsai et al., 2013). rEgAgB8/1 lipid composition depends on *Drosophila* metabolism and the conditions used during S2 cell culture. *Drosophila* can *de novo* synthesise non-essential saturated (C14:0, C16:0 and C18:0) and mono-unsaturated (C16:1, C18:1) FAs, which were found the most abundant FAs (free or esterified to phospholipids) in rEgAgB8/1. Furthermore, these FAs have been described as the principal FA species in the lipophorin, the major haemolymph lipoprotein of *Drosophila* (Matsuo et al., 2019). In addition, rEgAgB8/1 did not contain C20 and C22 PUFAs, which are absent in *Drosophila* because of the lack of the homologous Δ-6/Δ-5 desaturases, the key enzymes for their synthesis (Shen et al., 2010). Fruit flies also lack enzymes for synthesising C18:2 (Vrablik and Watts, 2013; Ziegler et al., 2015). Therefore C18:2 presence in rEgAgB8/1 phospholipids probably derives from the lipids uptaken by S2 cells during cell expansion, since expression was induced in a serum-free medium. The differences observed between the native and recombinant EgAgB made it worth characterising rEgAgB8/1 expressed in S2 cells in the presence of serum from hosts, to which the parasite is well-adapted.

Immunomodulatory studies were performed using a conventional model of macrophage activation by LPS, a TLR4 agonist previously used for EgAgB immunoregulatory studies (Riganò et al., 2007). Interestingly, LPS is usually present in the liver where the hydatid establishes and grows, as a result of its ability to cross the small intestinal barrier and traffic via the portal vein towards the liver (Akiba et al., 2020). Thus, a putative contribution of LPS to an inflammatory harmful environment for the parasite during infection cannot be discarded. Following previous reports, nEgAgB interfered with IL-6, IL-12p40 and IFN-β secretion triggered by LPS in macrophages *in vitro*. This suggests that nEgAgB affects the two main TLR4-downstream signalling pathways triggered by LPS since IL-6 and IL-12p40 secretion by macrophages has been mainly associated with MyD88/TIRAP/NF-ĸB while IFN-β secretion with TRAM/TRIF/IRF3 activation (Murphy et al., 1995; Fitzgerald et al., 2003; Yamamoto et al., 2003; Shen et al., 2008), respectively. The latter signalling pathway also contributes to sustaining cytokine responses via a late NF-ĸB activation (Ciesielska et al., 2021). In addition, nEgAgB interference with both signalling pathways would explain a reduction of IL-1β secretion and NO· generation; NF-ĸB activation is required for pro-IL-1β and NOS2 expression while TRAM/TRIF activation is particularly required for a robust NO· generation through IFN-β/STAT1 activation (Reis et al., 2011; Ciesielska et al., 2021; Yi et al., 2021). Unexpectedly, EgAgB did not inhibit LPS-induced CD86 and CD40 expression in BMDM, which mostly depends on TRAM/TRIF signalling in murine macrophages (Shen et al., 2008). This result suggests putative differences in the signalling mechanisms that govern the expression of cytokines and co-stimulatory receptors (CD86/CD40) in BMDM activated with LPS *in vitro*. However, EgAgB might establish complex interactions during LPS-induced BMDM activation, leading to interference with LPS recognition and/or signalling pathways but triggering in parallel alternative activation pathways that contribute to sustaining CD86/CD40 expression. For instance, plasma lipoprotein receptors might be involved in nEgAgB effects because binding studies showed that LDL and HDL competed with EgAgB for binding to THP-1 monocytes (Silva-Álvarez et al., 2016). Interestingly, rEgAgB mimicked all *in vitro* nEgAgB modulatory activities, supporting its utilisation as an alternative model for examining *in vitro* EgAgB effects on macrophages. Studies for deciphering cell signalling actions linked to EgAgB immunomodulatory effects in macrophages are in progress in our group.

Beyond the *in vitro* immunomodulatory actions on macrophages, nEgAgB and rEgAgB8/1 modulated the cytokine secretion and macrophage cell receptor expression induced by LPS in the mouse peritoneum. The regulation of the cytokine secretion by peritoneal cells, including a decrease in IL-6 and IL-12p40 (a trend in the case of nEgAgB) and an increase in IL-10 (a trend in the case of rEgAgB8/1), suggests that EgAgB can reduce the systemic and local inflammation triggered by LPS. Besides, nEgAgB diminished the cell surface expression of MHC-II in LPM and CD86 and CD40 in SPM, suggesting its ability to modulate the collaboration between effector T CD4+ lymphocytes and local macrophages in an LPS-induced inflammatory environment. Differences in LPM and SPM responses to LPS might explain the dissimilar regulatory actions of EgAgB on these macrophage populations. Cell surface MHC-II expression showed a 5-fold up-regulation in LPM while it was unchanged or slightly down-regulated in SPM. Cell surface CD86/CD40 expression exhibited between 5 and 2-fold increases in SPM compared to 1.5-fold increases in LPM. These observations agree with LPM’s description as a senescent macrophage population of the mouse peritoneum (Cassado et al., 2011). *In vivo*, EgAgB reduction of peritoneal IL-6 and IL-12p40 may be a consequence of its direct interaction with macrophages and dendritic cells according to our observations in BMDM and those described in human monocyte-derived dendritic cells (Riganò et al., 2007). On the other hand, EgAgB’s effects on cell receptor expression in LPM and SPM might arise from soluble mediators produced by other peritoneal cell populations in the presence of EgAgB. IL-10 would contribute to these putative indirect effects because mice treated with nEgAgB plus LPS showed higher levels compared to mice treated only with LPS, and IL-10’s ability to modulate the expression of MHC-II and/or CD86 in models of activated BMDM and peritoneal macrophages (Mittal et al., 2015). The fact that rEgAgB8/1 did not promote a significant IL-10 potentiation could explain why its modulatory actions on LPM and SPM were null or less robust. Which peritoneal cells are responsible for EgAgB-induced IL-10 potentiation needs to be studied; the involvement of macrophages and dendritic cells is uncertain since EgAgB did not enhance LPS-triggered IL-10 response in BMDM (this work) and dendritic cells (Riganò et al., 2007).

Another interesting finding was that nEgAgB and/or rEgAgB8/1 *per se* increased cell surface CD86 and CD40 expression in LPM and SPM. Intriguingly, the latter was not accompanied by an increased secretion of inflammatory cytokines in the peritoneal cavity. These observations suggest the potential of these lipoproteins to drive the acquisition of a particular phenotype by peritoneal macrophages *in vivo*. The mechanism involved in this effect requires further analysis. CD86 and CD40 increases were not observed upon EgAgB stimulation of BMDM *in vitro*. Differences in cellular properties between BMDM, LPM and SPM, and EgAgB interactions with other cells and/or soluble factors within the peritoneal cavity might, at least partially, explain such contrasting results. In the context of CE, induction of surface CD86 and CD40 in tissue macrophages by EgAgB might contribute to parasite immunomodulation by influencing macrophage interaction with tissue effector T CD4*^+^* lymphocytes, including FoxP3^+^ CD4^+^ regulatory T cells (Grezzi et al., 2024). Remarkably, this scenario would change in the presence of a pro-inflammatory signal, as shown in the presence of LPS. Indeed, as already discussed, under an inflammatory condition, EgAgB could diminish CD86 and/or CD40 expression in tissue macrophages, attenuating putative signalling actions by effector inflammatory T CD4+ lymphocytes and, thereby contributing to control macrophage toxic mechanisms and inflammation.

Our study also showed that rEgAgB8/1 mostly reproduced nEgAgB modulatory effects on the inflammatory activation of macrophages, although it appears to display lower activity *in vivo.* Therefore, this finding suggests that this apolipoprotein subunit plays a role in nEgAgB macrophage modulation. The EgAgB lipid moiety could be directly or indirectly (by influencing lipoprotein assembly and size) involved in the immunomodulatory actions, explaining the differences between the effects observed by the native and recombinant antigens. In this context, as mentioned above, the biochemical characterization of rEgAgB8/1 expressed in *Drosophila* cells in the presence of different lipid sources is worthy of further exploration; it could shed light on the lipoprotein composition and assembly as well as on the lipid contribution to EgAgB immunomodulatory properties.

Overall, this work supports that EgAgB might be involved in parasite-driven immunomodulation strategies in CE by interfering with the inflammatory activation of macrophages. *In vivo* studies, by silencing or enhancing EgAgB expression during CE, are needed to elucidate the biological significance of the EgAgB immunomodulatory effects observed in this work. To that end, CRIPSR/Cas9 targeted mutagenesis is a promising approach, although it still represents a challenge in worm parasites such as *Echinococcus* (Du et al., 2021). As an alternative strategy to address the physiological value of EgAgB modulatory actions on macrophages, the effect of nEgAgB/rEgAgB8/1 administration in other inflammatory models would be valuable. For this purpose, the murine model of inflammatory bowel disease might help to expand our studies according to the putative EgAgB anti-inflammatory actions recently described in this model (Bao et al., 2022).

## Data availability statement

The original contributions presented in the study are included in the article/**Supplementary Material**. Further inquiries can be directed to the corresponding author.

## Ethics statement

The animal study was approved by the Ethical Committee of the Parque Lecoq (Montevideo Municipal Zoo) and the Honorary Commission of Animal Experimentation (CHEA) of Facultad de Química (UdelaR), which belongs to the National Committee for Animal Experimentation of Uruguay (https://www.cnea.gub.uy/). The study was conducted in accordance with the local legislation and institutional requirements.

## Author contributions

AMFo: Formal analysis, Funding acquisition, Investigation, Methodology, Project administration, Validation, Visualization, Writing – original draft, Writing – review & editing. SL: Investigation, Validation. MF: Methodology, Supervision, Writing – review & editing. RA: Investigation, Writing – original draft. FC: Formal analysis, Investigation, Writing – review & editing. CV: Investigation. DW: Formal analysis, Investigation, Writing – review & editing. JJ: Investigation, Resources, Visualization, Writing – review & editing. GG-S: Resources. OP: Methodology, Resources. AG-T: Methodology, Formal analysis, Supervision, Writing – original draft. AMFe: Conceptualization, Formal analysis, Funding acquisition, Investigation, Methodology, Project administration, Writing – original draft, Writing – review & editing
