# Supplementary figures and images for: Modulatory actions of Echinococcus granulosus antigen B on macrophage inflammatory activation
Source: Front Cell Infect Microbiol. 2024 Mar 18;14:1362765. doi: 10.3389/fcimb.2024.1362765 (PMC10982386; doi:10.3389/fcimb.2024.1362765)

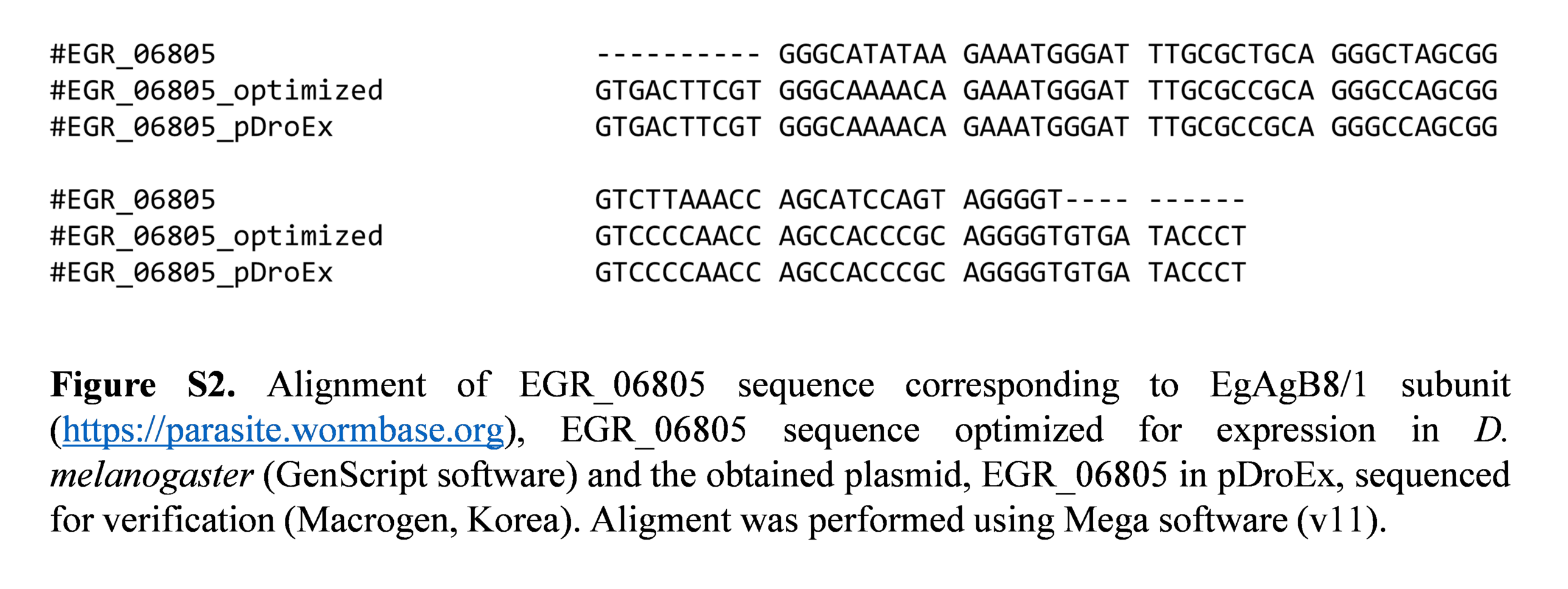

Supplement: Supplementary file 2 [file Image_2.jpeg]

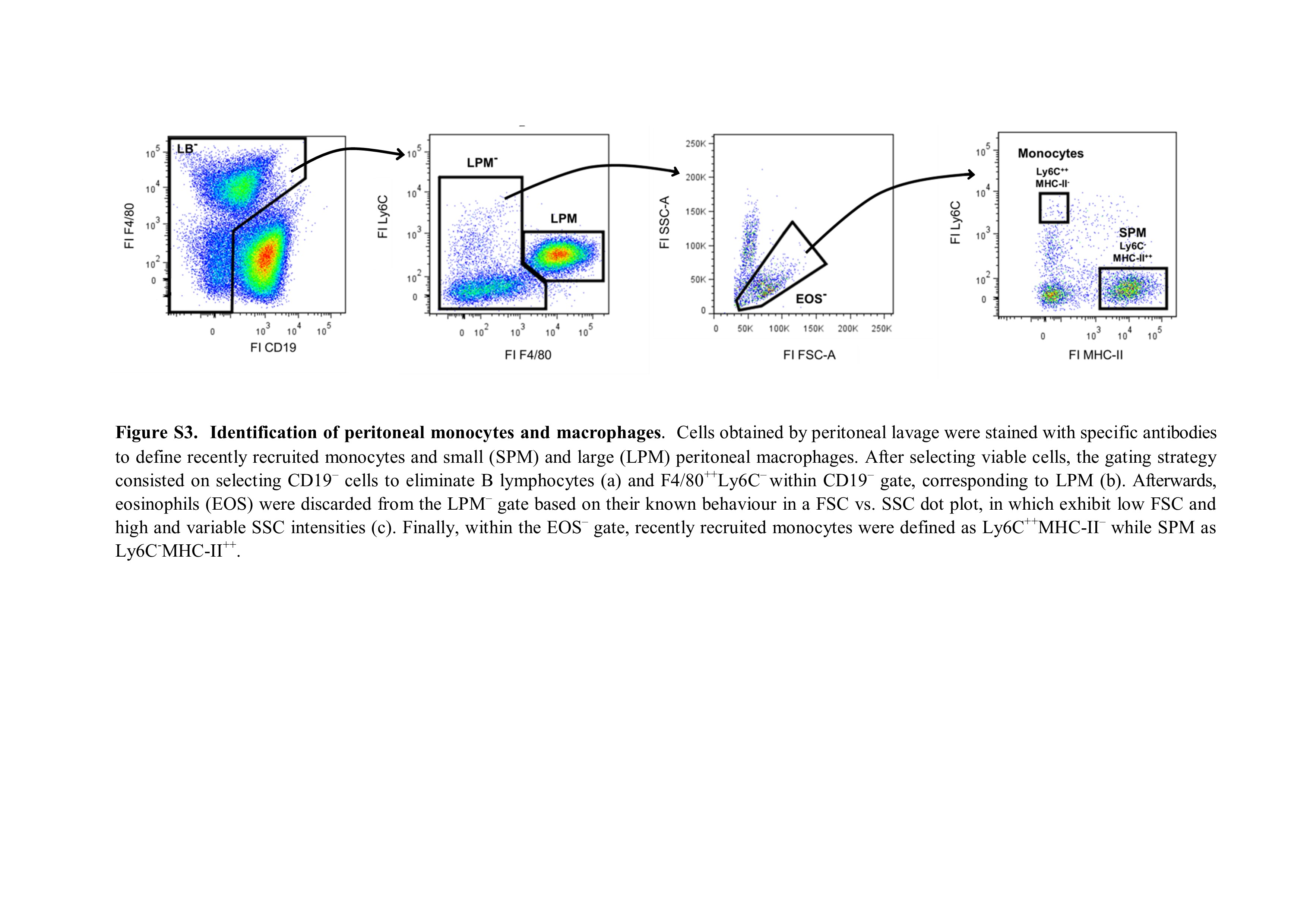

Supplement: Supplementary file 3 [file Image_3.jpeg]

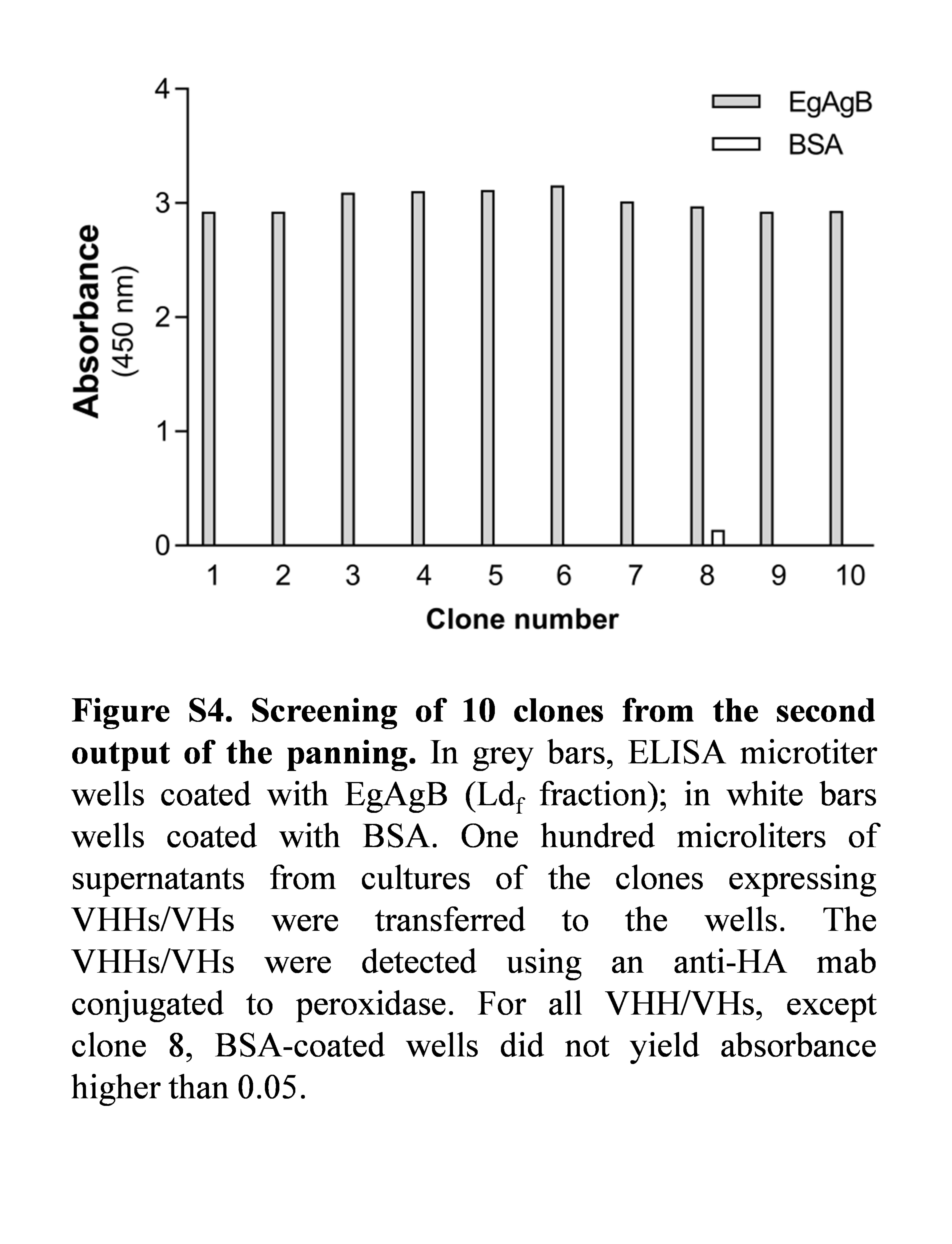

Supplement: Supplementary file 4 [file Image_4.jpeg]

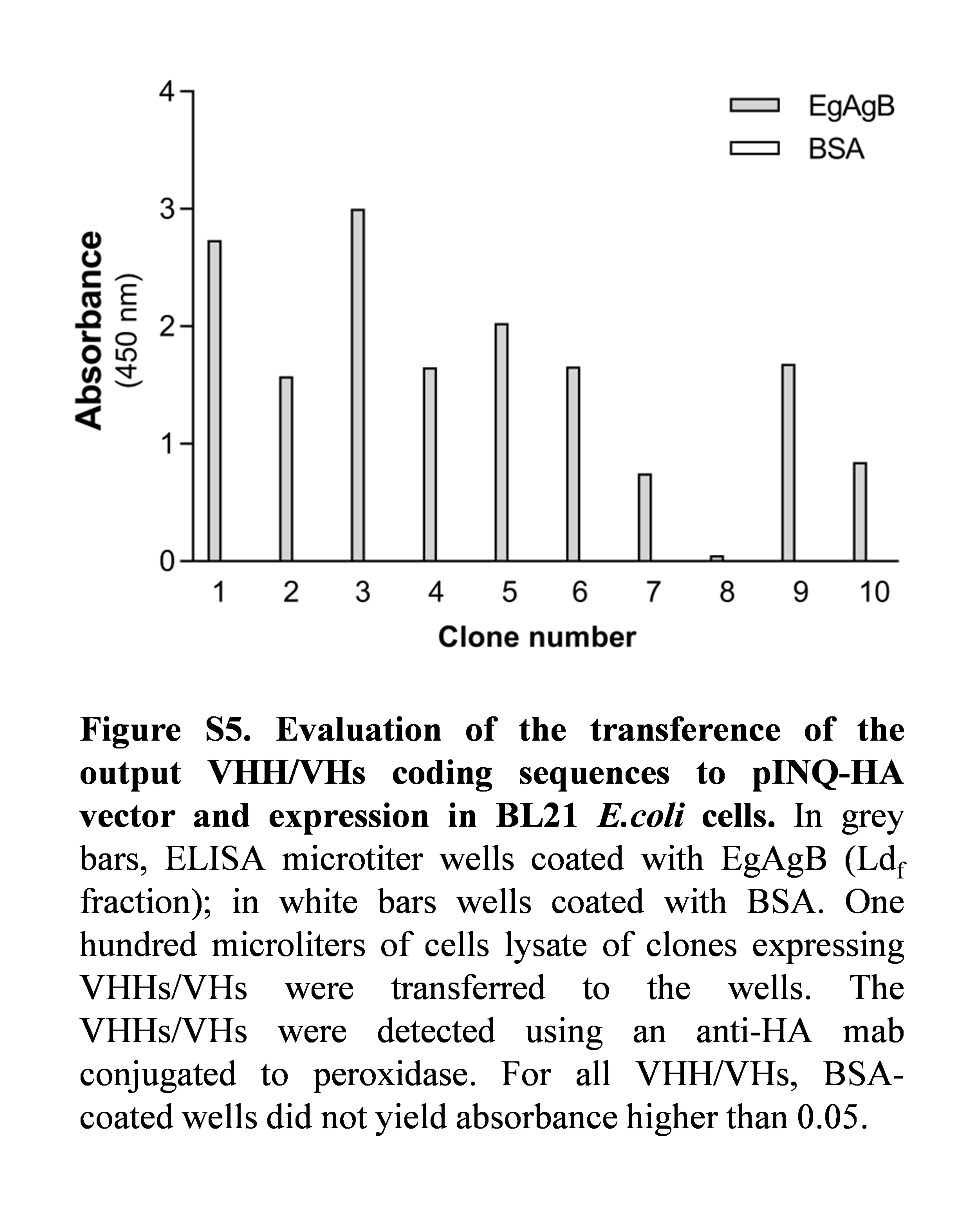

Supplement: Supplementary file 5 [file Image_5.jpeg]

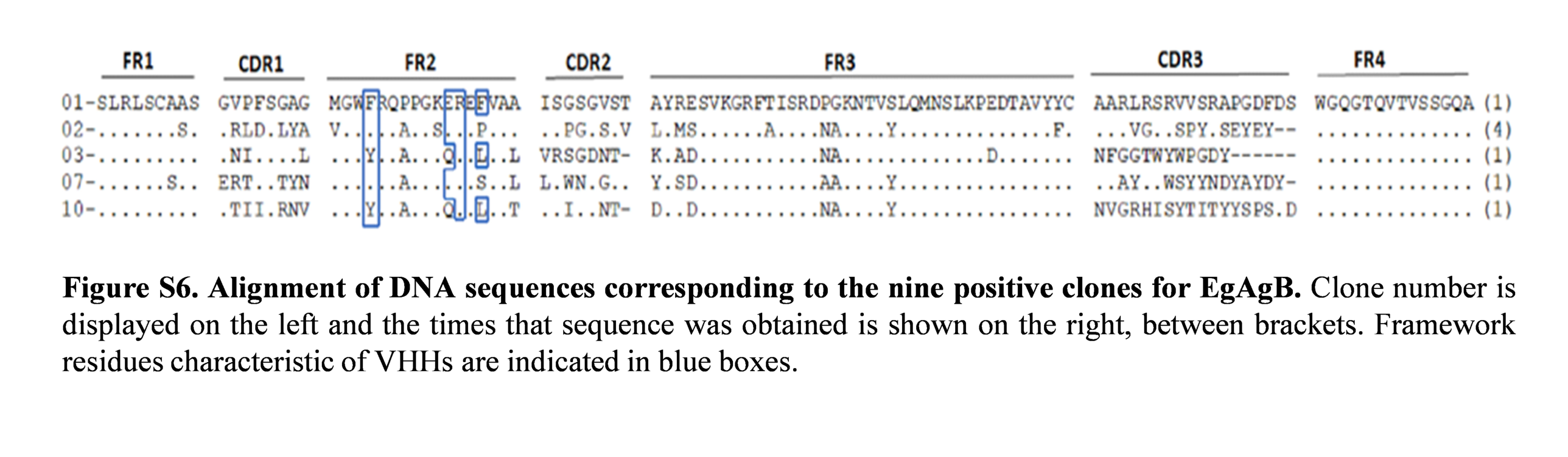

Supplement: Supplementary file 6 [file Image_6.jpeg]

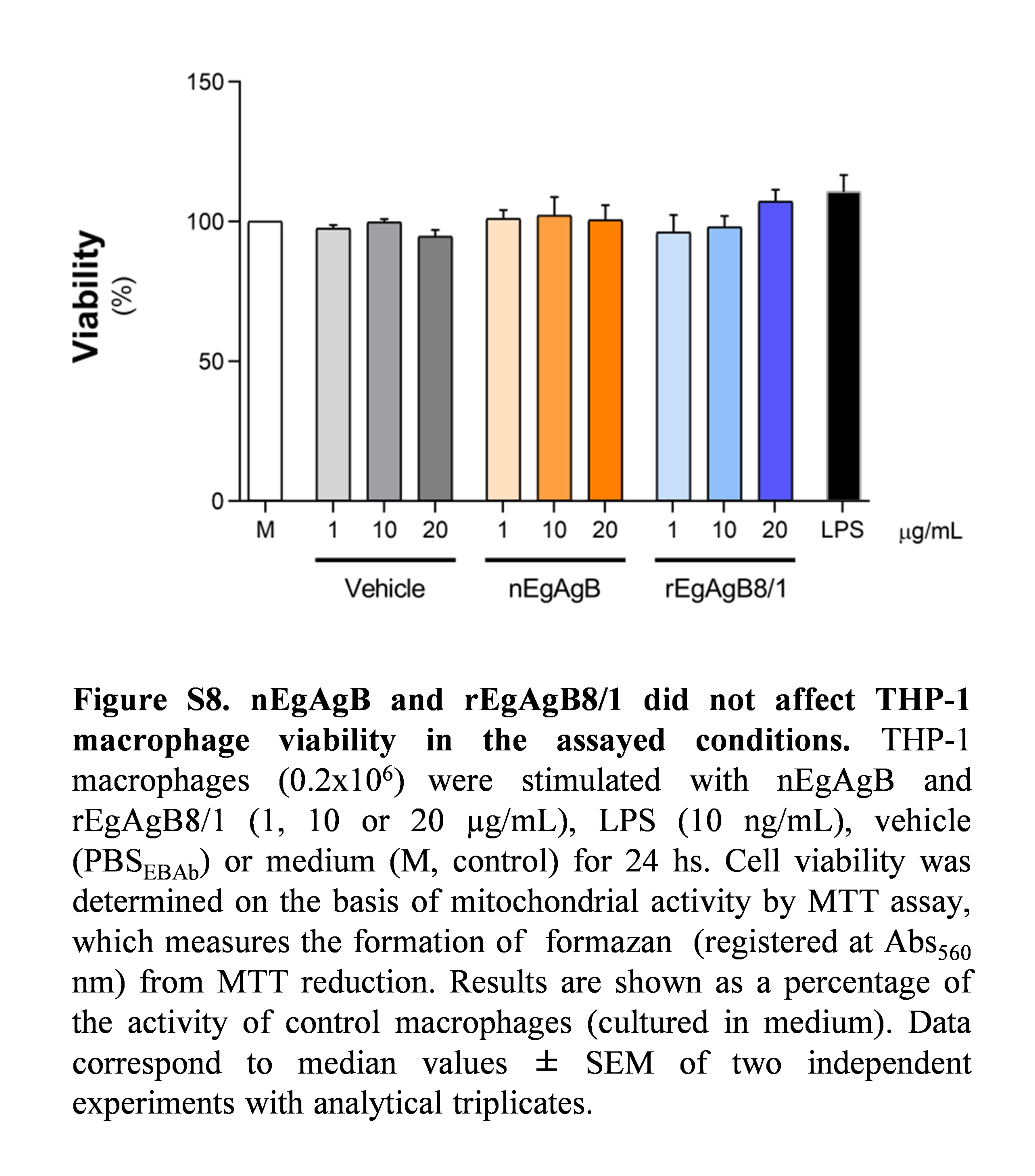

Supplement: Supplementary file 8 [file Image_8.jpeg]

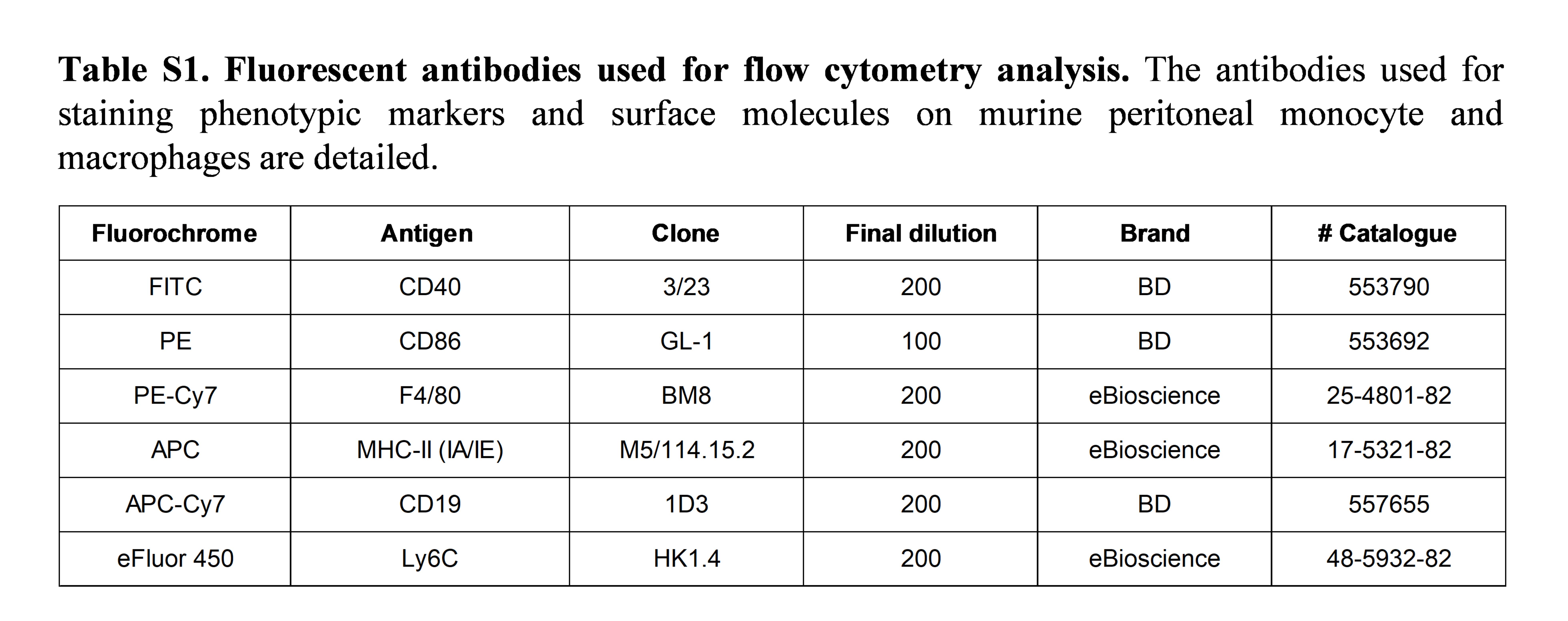

Supplement: Supplementary file 13 [file Image_13.jpeg]
